# Targeting *p21*‐High Senescent Kupffer Cells Nanotherapeutically Potentiates Antitumor Immunity in Advanced Hepatocellular Carcinoma with Portal Vein Tumor Thrombus

**DOI:** 10.1002/advs.76384

**Published:** 2026-07-02

**Authors:** Na Ta, Shuyi Wang, Boyan Zhang, Ning Liu, Yingchen Han, Aoran Liu, Ying Xu, Tingsong Chen, Ye Zhang, Qiuhua Luo, Tao Han

**Affiliations:** ^1^ Department of Medical Oncology The First Hospital of China Medical University Shenyang P. R. China; ^2^ Department of Neurology The Second Affiliated Hospital of Dalian Medical University Dalian P. R. China; ^3^ Department of Pharmacy The First Hospital of China Medical University Shenyang Liaoning P. R. China; ^4^ Department of Pancreatic and Biliary Surgery The First Affiliated Hospital of China Medical University Shenyang Liaoning P. R. China; ^5^ The First Laboratory of Cancer Institute The First Hospital of China Medical University Shenyang Liaoning P. R. China; ^6^ School of Medicine Southern University of Science and Technology Shenzhen P. R. China; ^7^ Department of Interventional Oncology Seventh People's Hospital of Shanghai University of Traditional Chinese Medicine Shanghai P. R. China

**Keywords:** combination immunotherapy, exosome‐based delivery, hepatocellular carcinoma, *p21*, portal vein tumor thrombus, senescent Kupffer cells, tumor microenvironment

## Abstract

Hepatocellular carcinoma (HCC) patients with portal vein tumor thrombus (PVTT) are associated with a significantly poor prognosis and limited treatment options. Single‐cell RNA sequencing (scRNA‐seq) has revealed senescent Kupffer cells (sKCs), which are characterized by high levels of *p21* expression and enriched in the tumor microenvironment (TME) of HCC‐PVTT. These sKCs exhibit a pronounced senescence‐associated secretory phenotype (SASP), promoting the proliferation and invasion of tumors and crosstalk with cancer‐associated fibroblasts. To target sKCs, a biomimetic nanodelivery system termed SKEV@AAV has been developed. This system comprises adeno‐associated virus (AAV) vectors carrying *p21*‐specific shRNA, encapsulated within sKCs‐derived exosomes (SKEV). SKEV@AAV effectively downregulated *p21*, suppressed SASP signaling, and disrupted pro‐tumor interactions between sKCs and cancer‐associated fibroblasts. In an orthotopic PVTT model, SKEV@AAV showed single‐agent antitumor activity and attenuated SASP‐associated inflammatory remodeling. Furthermore, the combination efficacy with anti‐PD‐1 was evaluated in a murine splenic liver‐metastasis model, where SKEV@AAV reduced tumor burden, enhanced CD8^+^ T‐cell infiltration, decreased regulatory T cells, and promoted memory T‐cell differentiation. Our findings reveal a pivotal role of sKCs in mediating immune suppression in HCC‐PVTT and provide a nanotherapeutic strategy that reverses sKCs' senescence, reprograms the TME, and ultimately enhances antitumor immune activation.

## Graphical abstract

Schematic illustration of a biomimetic SKEV@AAV platform targeting *p21*‐high senescent Kupffer cells (sKCs) in hepatocellular carcinoma (HCC) with portal vein tumor thrombus (PVTT). *p21*‐high sKCs accumulate in the PVTT microenvironment and establish an immunosuppressive, SASP‐rich environment that facilitates tumor cell proliferation and immune evasion. The SKEV@AAV platform delivers *p21*‐shRNA via sKCs‐derived exosomes to suppress SASP signaling, restore immune functions, and potentiate anti‐PD‐1 efficacy in HCC‐PVTT models.

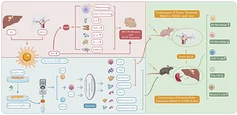

## 1 Introduction

Hepatocellular carcinoma (HCC) ranks among the leading causes of cancer‐related mortality worldwide, with its high mortality rate closely linked to the frequent occurrence of portal vein tumor thrombus (PVTT) in advanced stages [1]. PVTT not only limits treatment options but also accelerates intrahepatic spread and distant metastasis, serving as a critical determinant of poor prognosis in HCC patients [2]. Despite recent advancements in immunotherapy for various solid tumors, the response rate in advanced HCC patients with PVTT remains suboptimal, with underlying mechanisms still inadequately understood [3, 4]. The immune‐suppressive nature of the tumor microenvironment (TME) plays a central role in treatment resistance, with macrophages being the most abundant immune cell population, which directly influence the strength and persistence of the antitumor immune response, thereby representing a vital target for overcoming the challenges of HCC‐PVTT immunotherapy [5, 6].

HCC commonly arises in a background of chronic inflammatory liver disease, including viral hepatitis and steatotic liver disease, and persistent inflammatory or antigenic signaling is closely linked to immune dysfunction and T‐cell exhaustion, in which the PD‐1/PD‐L1 axis is an important component [7, 8]. Moreover, PVTT represents a distinctive intravascular metastatic niche characterized by hypoxia, metabolic stress, and profound immune remodeling, all of which have been implicated in PVTT progression [9]. Recent single‐cell studies further indicate that macrophage/monocyte populations are prominent components of the PVTT microenvironment and display transcriptional features distinct from those in the primary tumor. Collectively, these observations suggest that the microenvironment may be particularly permissive for the emergence and maintenance of senescence‐associated macrophage states, thereby providing a biological rationale for interrogating *p21*‐high senescent Kupffer cells (sKCs) in HCC with PVTT [10].

Recent advancements in single‐cell RNA sequencing (scRNA‐seq) technology have enabled unprecedented insights into the cellular heterogeneity and functional states within the TME [11]. Comprehensive scRNA‐seq analysis of HCC and PVTT tissue samples identified a population of sKCs exhibiting high expression of *CDKN1A* (*p21*), which are significantly enriched in the PVTT region, with their proportion increasing as the disease progresses. Intercellular communication analysis further suggests that sKCs engage in close interactions with HCC cells and cancer‐associated fibroblasts, indicating their potential role in promoting tumor immune evasion and disease progression by modulating TME components. Notably, emerging studies have highlighted the critical role of senescent macrophages in tumor development. For example, in a KRAS‐driven lung cancer model, the clearance of *p16^INK4a^*‐positive senescent macrophages significantly reduced tumor burden and enhanced immune surveillance [12]. These findings reinforce the notion that senescent immune cells are crucial regulators within the TME and may serve as promising therapeutic targets.

Building on the findings, targeting *p21*‐expressing sKCs may reverse the immune‐suppressive microenvironment in HCC‐PVTT, thereby enhancing the antitumor immune response. A gene drug delivery system, SKEV@AAV, was designed using sKCs‐derived exosomes as carriers to enable homologous targeting delivery. The core mechanism involves the targeted delivery of *p21*‐specific shRNA to selectively downregulate *p21* expression in sKCs, reshaping their senescent phenotype. Both in vitro and in vivo experiments systematically evaluated the efficiency of SKEV@AAV in silencing *p21* expression in sKCs, confirmed its inhibitory effects on senescence‐associated secretory phenotype (SASP) secretion, and explored its role in promoting T‐cell infiltration and activation. The synergistic antitumor effects of SKEV@AAV combined with PD‐1 blockade therapy were also assessed. Additionally, the study demonstrated that SKEV@AAV indirectly inhibits fibroblast secretion of MMP2 and MMP9 by reducing SASP secretion from sKCs, further diminishing the pro‐tumor characteristics of the TME and supporting the efficacy of combination immunotherapy. This study unveils a critical regulatory role of sKCs in the immune microenvironment of HCC‐PVTT and proposes senescence‐targeted macrophage reprogramming as a candidate liver‐directed immunotherapeutic strategy. By deeply analyzing the immune microenvironment of HCC and innovatively designing a drug delivery system with homologous targeting capabilities, the research provides new technological approaches and theoretical support to enhance HCC‐PVTT immunotherapy efficacy. These findings offer novel therapeutic strategies and experimental evidence for advanced HCC patients, particularly those with PVTT, with considerable potential for clinical translation.

## 2 Results

### 2.1 Single‐Cell Transcriptional Atlas Reveals the Enrichment of *p21‐*Highly Expressed sKCs in HCC with PVTT and Their Interactions in the TME

PVTT marks a critical clinical juncture in the progression of HCC, being closely associated with diseases invasiveness and limited treatment options. To systematically characterize the immune‐suppressive microenvironment underlying PVTT progression, Publicly available scRNA‐seq data from 20 clinical samples comprising non‐tumor liver (NTL, n = 8), primary tumor (PT, n = 10), and PVTT samples (n = 2) were integrated (Figure 1A). Following data harmonization and rigorous quality control, 19 cell clusters were identified and annotated into nine major cell types using established marker genes (Figure 1B; Figures S1 and S2). Notably, KCs were significantly enriched in PT and PVTT compared to NTL, suggesting their pivotal role in disease progression (Figure 1C).

**FIGURE 1:**
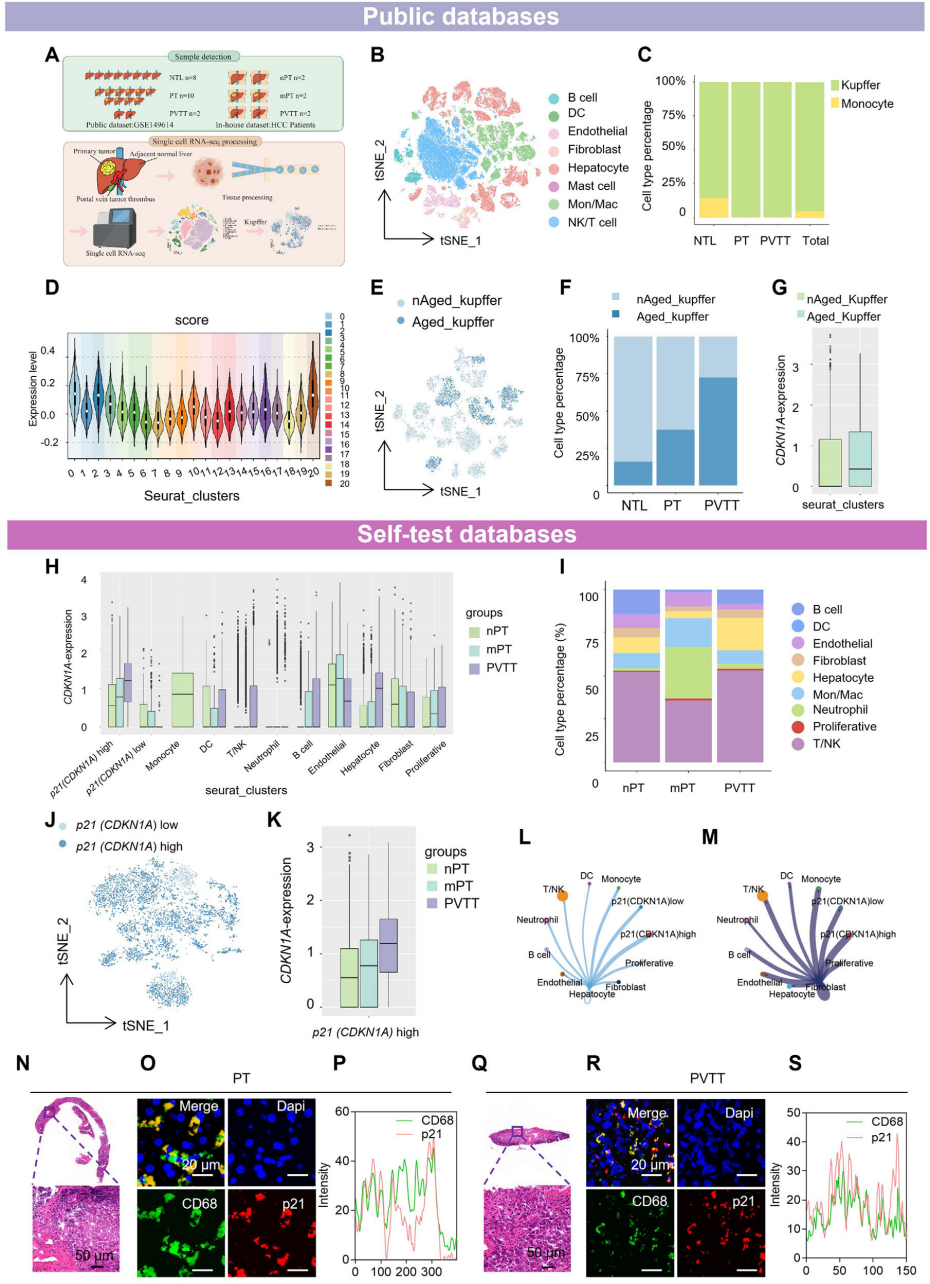
Single‐cell atlas and clinical data of HCC and PVTT. (A) Schematic representation of the overall study design. (B) t‐SNE plot showing the distribution of major cell populations, with each population highlighted by a distinct color code. (C) Proportional representation of Kupffer cell type in the samples, including NTL, PT, and PVTT. (D) Violin plot depicting the aging status of 20 KCs clusters, scored using the “AddModuleScore” algorithm. (E) t‐SNE map displaying the distribution of KCs subtypes. (F) Proportion of senescent KCs (Aged_Kupffer) and non‐senescent KCs (nAged_Kupffer) across different groups. (G) Boxplot illustrating *p21* expression across various KCs types. (H) t‐SNE visualization of major cell population distribution in different groups, with each population identified by a specific color code. (I) Proportion of all cell types in the samples, which include nPT, mPT, and PVTT. (J) t‐SNE visualization of KCs subpopulations. (K) Boxplot demonstrating *p21* expression in *p21‐*high KCs across different groups. (L,M) Cell‐cell communication between *p21‐*high KCs and hepatocytes, as well as fibroblasts. (N) H&E staining of PT from patients, scale bar: 50 µm. (O,P) Representative confocal images showing co‐localization of CD68 (green) and p21 (red) in HCC tissue, with quantitative analysis. Nuclei were counterstained with DAPI (blue). Scale bar: 20 µm. (Q) H&E staining of PVTT from patients, scale bar: 50 µm. (R,S) Representative confocal images showing co‐localization of CD68 (green) and p21 (red) in tumor thrombus tissue, along with quantitative analysis. Nuclei were counterstained with DAPI (blue). Scale bar: 20 µm.

Further subpopulation analysis of KCs revealed 20 transcriptionally distinct subclusters (Figure S3). Applying established senescence gene signatures and the AddModuleScore algorithm, a unique subcluster of sKCs was identified, termed “Aged_Kupffer”, which exhibited significantly elevated expression of the classic senescence marker *p21* (Figure 1D,E,G) [13]. The proportion of sKCs progressively increased in PT and PVTT compared to NTL (Figure 1F). Given the limited sample size of the initial discovery cohort, this observation should be regarded as a hypothesis finding that warrants further validation. Therefore, to validate the aforementioned findings, we employed an independent validation cohort consisting of six HCC patients, including non‐metastatic primary tumor (nPT), metastatic primary tumor (mPT), and PVTT tissues. Consistent results were obtained, thereby corroborating our initial observations. The abundance of *p21*‐positive senescent KCs consistently increased with disease progression, reaching its peak in PVTT (Figure 1H–K; Figures S4–S6).

To assess their functional significance, copy number variation (CNV) analysis was performed, confirming the presence of a hepatic malignant cell cluster (Figure S7). Intercellular communication analysis revealed that *p21*‐positive senescent KCs serve as core nodes interacting with hepatic malignant cells and fibroblasts, highlighting their pivotal role in remodeling the TME (Figure 1L,M) [14, 15, 16].

Clinical validation using paired patient samples reinforced these findings. Hematoxylin and eosin (H&E) staining clearly depicted the histopathological structures of NTL, PT, and PVTT (Figure 1N,Q; Figure S8). Notably, immunofluorescence co‐staining demonstrated significant co‐localization of p21 and CD68^+^ KCs in PT and PVTT, in contrast to the weak signal in NTL (Figure 1O,P,R,S; Figure S9). These results support the hypothesis that *p21*‐driven KCs senescence plays a critical role in TME dysregulation and clinical invasiveness.

### 2.2 Design and Characterization of a Drug Delivery System Targeting sKCs with High *p21* Expression

To efficiently and precisely reverse sKCs with high *p21* expression and inhibit their pro‐tumor proliferative activity [17], an adeno‐associated virus (AAV) carrying *p21*‐shRNA and two exosome‐based delivery systems were developed (Tables S1 and S2): (1) KEV@AAV, where exosomes derived from young KCs encapsulate AAV‐*p21* shRNA; (2) SKEV@AAV, where exosomes derived from doxorubicin (DOX)‐induced sKCs encapsulate AAV‐*p21* shRNA. Optimal senescence induction was achieved through gradient DOX treatment (30–150 nmol/mL), with 80 nmol/mL selected as the ideal concentration to induce maximal senescence while minimizing cytotoxicity (Figure S10). Western blotting further validated the upregulation of p21 in DOX‐treated cells, with protein levels significantly higher than those in KCs, demonstrating successful senescence modeling (Figure S11). As expected, sKCs exhibited a markedly reduced proportion of EdU‐positive cells compared with KCs, indicating a substantial decrease in DNA synthesis and supporting the presence of proliferative arrest, a key hallmark of cellular senescence (Figure S12). Exosomes were then extracted from KCs and sKCs by ultracentrifugation, yielding KEV and SKEV, respectively. These exosomes were mixed with AAV‐*p21* shRNA and extruded through a polycarbonate membrane to produce the drug‐loaded vesicles KEV@AAV and SKEV@AAV (Figure 2A).

**FIGURE 2:**
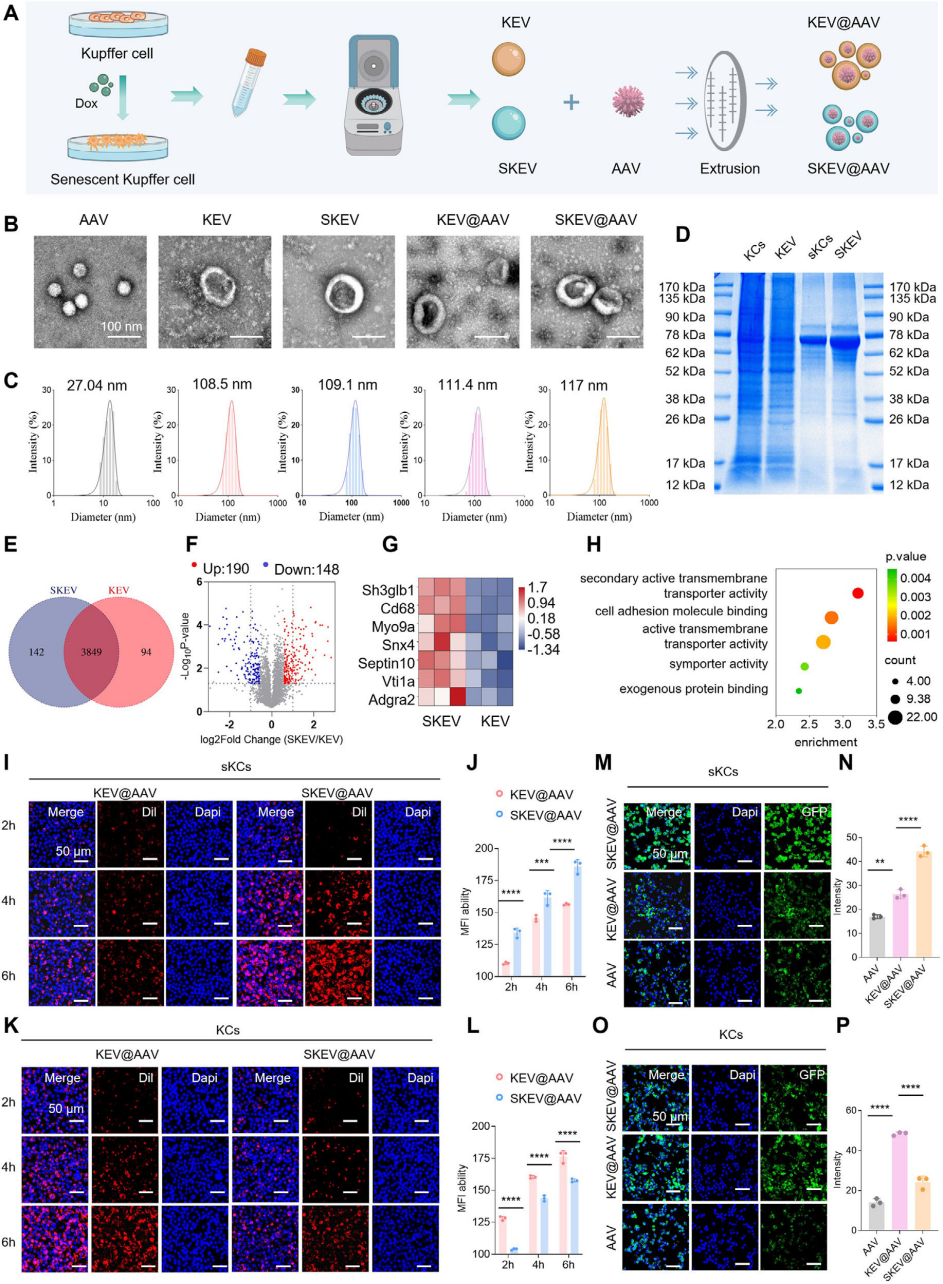
Synthesis and ex vivo characterization of SKEV@AAV. (A) Schematic diagram illustrating the synthesis process of AAV‐loaded KEV@AAV and SKEV@AAV. (B,C) TEM images and particle size distribution curves of AAV, KEV, SKEV, KEV@AAV, and SKEV@AAV. (D) SDS‐PAGE protein analysis of KCs, KEV, sKCs, and SKEV (n = 3). (E) Venn diagram showing overlapping and differentially expressed proteins between SKEV and KEV groups (n = 3). (F) Volcano plot displaying significantly differentially expressed proteins between SKEV and KEV (190 upregulated and 148 downregulated, *p* < 0.05). (G,H) Heatmap and functional enrichment analysis of transmembrane transport‐related differentially expressed proteins between SKEV and KEV groups. (I) Confocal laser scanning microscopy (CLSM) illustrating the uptake of EVs by sKCs after incubation with Dil (red)‐labeled KEV@AAV and SKEV@AAV for 2, 4, and 6 h. Nuclei were counterstained with DAPI (blue) (scale bar: 50 µm). (J) Quantitative analysis of the mean fluorescence intensity (MFI) of EVs uptake by sKCs at different incubation times (n = 3). (K) CLSM illustrating the uptake of EVs by KCs after incubation with Dil (red)‐labeled KEV@AAV and SKEV@AAV for 2, 4, and 6 h. Nuclei were counterstained with DAPI (blue) (scale bar: 50 µm). (L) Quantitative analysis of the MFI of EVs uptake by KCs at different incubation times (n = 3). (M) CLSM showing the uptake of AAV by sKCs after incubation with GFP (green)‐labeled KEV@AAV and SKEV@AAV. Nuclei were counterstained with DAPI (blue) (scale bar: 50 µm). (N) Quantitative analysis of the MFI of KEV@AAV and SKEV@AAV infection by sKCs (n = 3). (O) CLSM showing the uptake of AAV by KCs after incubation with GFP (green)‐labeled KEV@AAV and SKEV@AAV. Nuclei were counterstained with DAPI (blue) (scale bar: 50 µm). (P) Quantitative analysis of the MFI of KEV@AAV and SKEV@AAV infection by KCs (n = 3). Data are presented as mean ± SD. ^*^ *p* < 0.05, ^**^ *p* < 0.01, ^***^ *p* < 0.001, and ^****^ *p* < 0.0001.The infection efficiency of KEV@AAV and SKEV@AAVwas evaluated in KCs and sKCs over 48 h.

Regarding the quality control of the AAV preparation used for SKEV@AAV fabrication, transmission electron microscopy (TEM) negative staining analysis was performed to assess the full/empty capsid ratio. Representative images showed full AAV capsids (n = 152) and empty capsids (n = 7), yielding a full/empty ratio of 95.60% full particles (Scale bar: 100 nm) (Figure S13). This high proportion of full capsids indicates that the AAV preparation was of sufficient quality for subsequent encapsulation and functional studies. We next characterized the final KEV@AAV and SKEV@AAV complexes. TEM confirmed the typical icosahedral structure of AAV, with both vesicles exhibiting the characteristic cup‐shaped morphology of exosomes (Figure 2B). Dynamic light scattering (DLS) showed that the average particle size of KEV@AAV was 111.4 nm and SKEV@AAV was 117.0 nm (Figure 2C; Table S3). Western blotting confirmed the high expression of exosome marker proteins such as TSG101, CD63, and CD81 in all exosome samples, while AAV, KCs, and sKCs lysates showed no corresponding bands (Figure S14). Quantitative PCR indicated that the AAV encapsulation efficiency was 97.6% for KEV@AAV and 98.7% for SKEV@AAV (Figure S15). CLSM revealed strong colocalization of Dil and GFP‐labeled AAV, confirming effective encapsulation (Figure S16). The zeta potential of AAV was −5.4 mV; after encapsulation, the zeta potentials of KEV@AAV and SKEV@AAV were −15.17 mV and −12.63 mV, respectively (Figure S17). Cytotoxicity study performed using an MTT assay revealed negligible cytotoxicity at the therapeutic dose of AAV, which was selected for subsequent experiments (Figure S18). H&E staining revealed preserved hepatic lobular architecture in both BALB/c nude and C57BL/6 mice. No obvious hepatocellular necrosis, inflammatory infiltration, sinusoidal congestion, steatosis, or structural disruption was observed. These results indicate that SKEV@AAV did not cause detectable histopathological liver injury in healthy mice under the tested conditions (Figure S19). SDS‐PAGE analysis confirmed that the exosome protein profile closely matched that of its parent cells, suggesting that homologous membrane signaling enables directional homing, which may facilitate efficient and specific homologous targeting delivery (Figure 2D).

To investigate the functional differences between SKEV and KEV, quantitative proteomics based on LC‐MS/MS was conducted. The Venn diagram revealed 4085 common proteins between the two groups, with 142 proteins unique to SKEV and 94 unique to KEV (Figure 2E). The volcano plot, using a threshold of *p* < 0.05 and |log2 fold change| > 1, identified 338 differentially expressed proteins, with 190 upregulated and 148 downregulated in SKEV (Figure 2F). By analyzing differential proteins within SKEV and KEV, we identified key candidates with elevated abundance in SKEV, demonstrating their distinct advantages in enhancing cell adhesion, endocytosis, uptake efficiency, and transmembrane transport (heatmap) (Figure 2G) [18, 19, 20, 21]. Further Gene Ontology (GO) enrichment analysis confirmed that these proteins were significantly enriched in pathways related to cell adhesion molecule binding, secondary active transmembrane transporter activity, and cotransporter function (Figure 2H). These results indicate that SKEV possesses notable advantages in membrane transport and cell recognition.

To assess the intracellular delivery efficiency of SKEV@AAV and KEV@AAV, sKCs and KCs were incubated with Dil‐labeled vesicles. CLSM revealed that, in sKCs, the intracellular red fluorescence of the SKEV@AAV group was significantly stronger than that of the KEV@AAV group at 2, 4, and 6 h, with the fluorescence signal increasing over time (Figure 2I,J). In contrast, in KCs, KEV@AAV uptake was higher than SKEV@AAV, indicating the homologous targeting of EV formulations to their parent cells (Figure 2K,L). In the GFP‐AAV infection experiment, confocal images showed the intracellular distribution of green fluorescence. In the sKCs group, SKEV@AAV exhibited a stronger GFP signal after 48 h infection (Figure 2M,N), while in normal KCs, the infection efficiency of KEV@AAV was higher than SKEV@AAV (Figure 2O,P). To strengthen the evidence for homologous targeting in vitro, we have conducted the proposed competitive uptake experiment as follows: After co‐culture of normal KCs and sKCs, Dir‐labeled KEV@AAV and Dil‐labeled SKEV@AAV was added to the mixed cell population. At three time points (2, 4, and 6 h), flow cytometry was performed to detect the uptake of KEV@AAV‐Dir and SKEV@AAV‐Dil by p21‐positive (sKCs) and p21‐negative (KCs) cells, respectively. The results demonstrated that SKEV@AAV exhibited significantly higher uptake in p21‐positive sKCs compared to p21‐negative KCs at all tested time points, with the most pronounced difference observed at 6 h. These findings confirm that SKEV@AAV preferentially targets sKCs even in the presence of competing normal KCs (Figure S20). These findings provide a solid foundation for subsequent in vitro and in vivo functional studies.

### 2.3 SKEV@AAV Precisely Reverses Senescence and Blocks the Pro‐Tumor Function of sKCs

To systematically investigate the pro‐tumor function of sKCs in the TME [22] and the therapeutic efficacy of the *p21*‐targeted delivery system (SKEV@AAV), an integrated platform comprising Transwell co‐culture and EdU dual labeling was constructed (Figure 3A). Initially, sKCs were co‐cultured with Hepa1‐6 HCC cells, while sTHP‐1 cells were co‐cultured with CSQT‐2 and HuH7 cells, with EdU experiments confirming that the proliferation rates of Hepa1‐6 (Figure 3B,C), CSQT‐2 (Figure 3D,E), and HuH7 cells (Figure S21) were significantly increased [23]. Bulk RNA sequencing further showed that after co‐culture with sTHP‐1 cells, the gene pathways related to “DNA replication” and “cell cycle processes” in tumor thrombus cells were significantly upregulated, indicating active cell division and enhanced DNA replication efficiency, which likely drives the rapid proliferation of these cells. Additionally, pathways associated with “chromosome separation” were also upregulated, further reflecting increased cell division activity. Furthermore, the upregulation of “positive regulation of cell cycle process” and “regulation of cell cycle phase transition” suggested activation of cell cycle regulatory mechanisms, thereby providing additional support for tumor thrombus cell proliferation (Figure 3F). These findings imply that sTHP‐1 cells create a “division‐ready” microenvironment for tumor thrombus cells by activating DNA replication and cell cycle processes.

**FIGURE 3:**
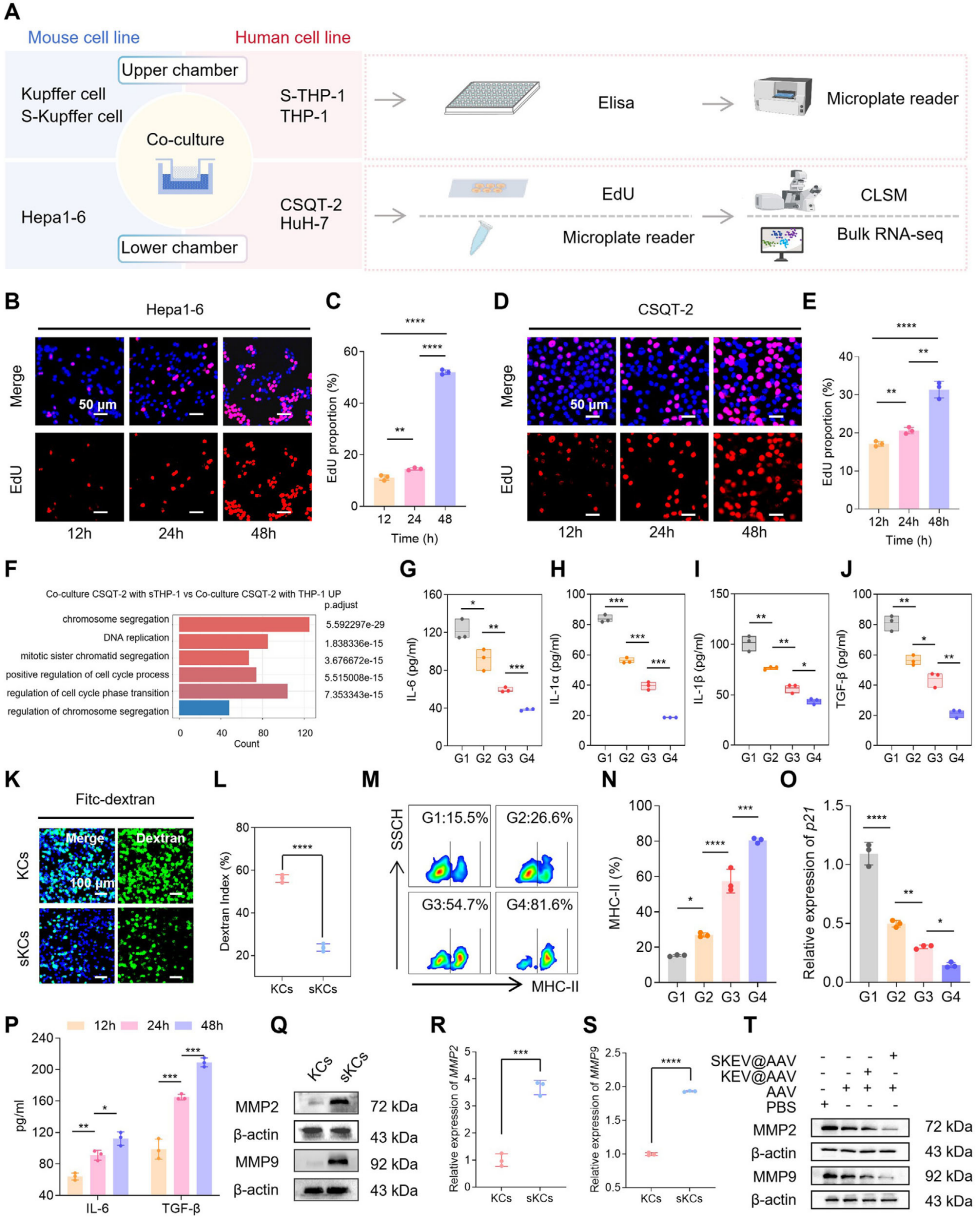
In vitro pharmacological evaluation of SKEV@AAV formulation. (A) A flowchart diagram illustrating the combination of Transwell co‐culture with EdU proliferation assay and Bulk RNA‐seq to assess the effects of sKCs on target cells. (B) The EdU proliferation assay confirmed that, after co‐culturing sKCs with Hepa1‐6 HCC cells, confocal images showed Hepa1‐6 cell proliferation. (C) Quantitative analysis of the MFI of Hepa1‐6 cell proliferation after co‐culturing sKCs with Hepa1‐6 HCC cells (n = 3). (D) After co‐culturing THP‐1 cells and sTHP‐1 cells with CSQT‐2 tumor thrombus cells, confocal images showed CSQT‐2 cell proliferation. (E) Quantitative analysis of CSQT‐2 cell proliferation after co‐culture with THP‐1 cells or sTHP‐1 cells (n = 3). (F) After co‐culturing CSQT‐2 with THP‐1 cells and sTHP‐1 cells, respectively, Bulk RNA‐seq was used to explore the pro‐proliferative mechanism of sTHP‐1 cells on CSQT‐2. (G–J) After the addition of PBS (G1), AAV (G2), KEV@AAV (G3), and SKEV@AAV (G4) to the supernatant of sKCs, ELISA quantified the levels of SASP factors (IL‐1α, IL‐6, IL‐1β, TGF‐β) in the supernatant of each treated group of sKCs (n = 3). (K) CLSM showed that the endocytic activity of sKCs was significantly lower than that of KCs. (L) Quantitative analysis of the MFI of endocytic activity in KCs and sKCs (n = 3). (M) After the addition of PBS (G1), AAV (G2), KEV@AAV (G3), or SKEV@AAV (G4) to the supernatant of sKCs, flow cytometry was used to detect the expression of antigen‐presenting molecules in each treated group of sKCs. (N) Quantitative analysis of the expression of antigen‐presenting molecules in each treated group (n = 3). (O) After the addition of PBS (G1), AAV (G2), KEV@AAV (G3), or SKEV@AAV (G4) to the supernatant of sKCs, qPCR showed the mRNA expression of *p21* in each treated group (n = 3). (P) ELISA was used to detect the levels of IL‐6 and TGF‐β in the lower chamber culture medium after co‐culturing sKCs with NIH/3T3 (n = 3). (Q) Western blot analysis was used to detect differences in the protein levels of MMP2 and MMP9 in NIH/3T3 cells stimulated by sKCs conditioned medium (n = 3). (R,S) qPCR was used to detect differences in the transcription levels of *MMP2* and *MMP9* in NIH/3T3 cells stimulated by sKCs conditioned medium (n = 3). (T) In the sKCs‐NIH/3T3 co‐culture system, after the addition of PBS (G1), AAV (G2), KEV@AAV (G3), or SKEV@AAV (G4) to the upper chamber, Western blot analysis was used to detect differences in MMP2 and MMP9 expression in NIH/3T3 of each treated group (n = 3). Data are presented as mean ± SD. ^*^ *p* < 0.05, ^**^ *p* < 0.01, ^***^ *p* < 0.001, and ^****^ *p* < 0.0001.

Moreover, SKEV@AAV was used to intervene in the senescent phenotype of sKCs. In the co‐culture system of tumor cells and sKCs, SKEV@AAV successfully inhibited tumor cell proliferation by reversing senescence (Figures S22–S24). To demonstrate that the therapeutic effect is mediated predominantly through senescent sKCs rather than through direct suppression of tumor cells. We performed additional control experiments in which CSQT‐2 and Hepa1‐6 tumor cells were treated directly with STEV@AAV or SKEV@AAV in the absence of sTHP‐1 cells or sKCs. Cell proliferation was evaluated by EdU incorporation, and *p21* mRNA expression was measured by qPCR. Finally, direct treatment with STEV@AAV or SKEV@AAV did not significantly change the percentage of EdU‐positive CSQT‐2 or Hepa1‐6 cells compared with PBS‐treated controls. Consistently, qPCR analysis showed that *p21* mRNA levels in both tumor cell lines were not significantly altered after direct STEV@AAV or SKEV@AAV treatment (Figures S25 and S26). These results indicate that STEV@AAV or SKEV@AAV does not directly suppress tumor‐cell proliferation or directly downregulate *p21* expression in CSQT‐2 or Hepa1‐6 cells under our experimental conditions.

To characterize the baseline secretory profile, we performed a time‐course ELISA analysis of conditioned medium harvested from DOX‐induced sKCs at the indicated time points. Representative SASP‐associated factors, including IL‐6, IL‐1α, IL‐1β, and TGF‐β, showed progressive accumulation in the supernatant over the 2–6 h time window (Figure S27). Building on the above characterization of the baseline SASP secretory profile, we further examined the effect of SKEV@AAV treatment on SASP secretion. ELISA results indicated that, compared to the PBS group, the levels of IL‐1α, IL‐6, IL‐1β, and TGF‐β in the supernatant of SKEV@AAV‐treated sKCs were significantly reduced (Figure 3G–J). Subsequently, differences in endocytic and antigen‐presenting functions between KCs and sKCs were compared. Confocal imaging revealed that sKCs exhibited significantly reduced endocytic activity compared to KCs (Figure 3K,L; Figure S28). Flow cytometry using MHC‐II as a marker further confirmed that sKCs also had significantly impaired antigen‐presenting function (Figure S29). Flow cytometric analysis showed that, compared with co‐culture with normal KCs, co‐culture with sKCs resulted in a significant reduction in the proportion of CD8^+^ T cells and a significant increase in the proportion of CD4^+^Foxp3^+^ Treg cells (Figure S30). These results collectively demonstrate that, due to senescence, sKCs lose their immune surveillance capability, and their “pro‐tumor” phenotype is associated with defects in antigen presentation. To explore whether SKEV@AAV could awaken the functional capabilities of sKCs, PBS (G1), AAV (G2), KEV@AAV (G3), or SKEV@AAV (G4) was added to the supernatant of sKCs. Compared to the PBS group, the SKEV@AAV group showed an 81.6% increase in MHC‐II expression (Figure 3M,N), an 86% decrease in *p21* mRNA expression (Figure 3O), and a 7‐fold increase in endocytic function (Figure S31). In addition, to directly evaluate whether treated sKCs regain proliferative capacity, we performed an EdU incorporation assay on sKCs themselves. The EdU data showed that SKEV@AAV treatment significantly increased the proportion of EdU‐positive sKCs compared with PBS, SKEV, AAV, and KEV@AAV. However, SKEV alone was insufficient to reproduce the effect of SKEV@AAV on *p21* downregulation or EdU incorporation (Figure S32). Similarly, qPCR indicated that SKEV alone did not substantially reduce *p21* expression in sKCs (Figure S33).

CAFs represent the most critical components within the TME of HCC and are primarily derived from hepatic stellate cells, promoting HCC progression through extracellular matrix (ECM) remodeling. NIH/3T3 fibroblasts were transformed into CAFs by treatment with recombinant mouse TGF‐β1 for 48 h. Next, the paracrine effect of sKCs on fibroblasts was evaluated. When sKCs were co‐cultured with NIH/3T3 cells, the concentrations of IL‐6 and TGF‐β in the lower chamber culture medium increased in a time‐dependent manner, reaching 1.76 and 2.11 times at 12 h at 48 h, respectively (Figure 3P). Western blot and qPCR results consistently showed that sKCs‐conditioned medium and sTHP‑1‑conditioned medium significantly upregulated the protein and transcriptional levels of MMP2/MMP9 in NIH/3T3 (Figure 3Q–S; Figures S34 and S35) and LX‐2 cells (Figures S36–S38), respectively.

In summary, sKCs not only drive tumor cells to enter a state of rapid proliferation but also further remodel the matrix and promote tumor invasion through fibroblasts [24]. To determine if reversing the senescent phenotype of sKCs can suppress their tumor promoting proliferative effects, the four formulations were introduced into the co‐culture system to assess their effects on MMP expression and cytokine release. Western blot (Figure 3T; Figures S39–S42) and qPCR (Figures S43 and S44) analysis showed that SKEV@AAV and STEV@AAV could significantly downregulate MMP2/MMP9 expression in NIH/3T3 and LX‐2 cells, respectively, and reduced the secretion of IL‐6 and TGF‐β (Figure S45). In addition, we performed additional rescue experiments in the sKCs–CAF transwell co‐culture system under SKEV@AAV treatment by exogenously supplementing key SASP‐related factors, including IL‐6, IL‐1α, IL‐1β, and TGF‐β, either individually or in combination. SKEV@AAV markedly reduced MMP2 and MMP9 expression in CAFs compared with the PBS control group. Importantly, supplementation with the combined cytokine cocktail produced the most evident rescue effect. In contrast, IL‐6, IL‐1α, IL‐1β, or TGF‐β alone showed only limited reversal under these conditions (Figure S46).

### 2.4 The Spatiotemporal Distribution and Targeting Enrichment of SKEV@AAV in Tumor Thrombus‐Bearing Mice

To investigate the in vivo targeting efficiency of SKEV@AAV, we assessed its distribution in nude mice bearing tumor thrombi. Immunofluorescence analysis revealed that CD68^+^ cells in the tumor thrombus region exhibited significant colocalization with the elevated p21 signal, whereas p21 expression was weak in normal liver tissue, thus confirming that CD68^+^ cells in the tumor thrombus with high p21 expression might be potential targets (Figure 4A,B).

**FIGURE 4:**
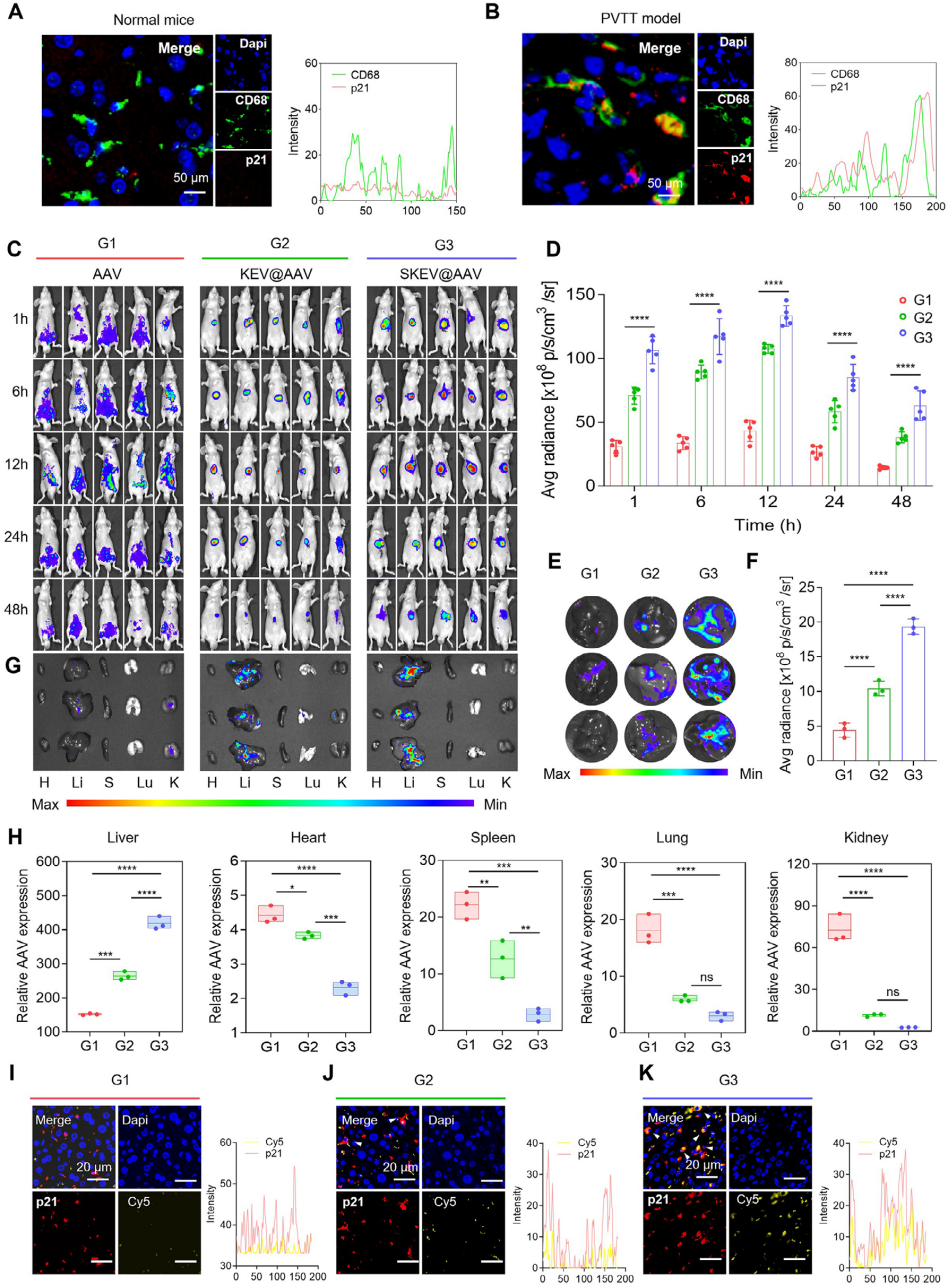
Distribution of SKEV@AAV in the tumor thrombus model. (A,B) Immunofluorescence staining was performed on normal liver tissue and tumor thrombus tissue, displaying cell nuclei (DAPI, blue), CD68^+^ cells (green), and p21 (red). Scale bar: 50 µm, (n = 3). (C) IVIS imaging demonstrated the distribution of Cy5‐labeled vectors in nude mice at 1, 6, 12, 24, and 48 h after intravenous injection. (D) Quantitative analysis of fluorescence intensity in nude mice at 1, 6, 12, 24, and 48 h (n = 5). (E) At 48 h, IVIS imaging showed the fluorescence intensity of the formulations in ex vivo liver tissue. (F) Quantitative analysis of fluorescence intensity of the formulations in ex vivo liver tissue at 48 h (n = 3). (G) At 48 h, ex vivo IVIS imaging of major organs (heart, liver, spleen, lung, kidney) in mice from each treatment group to assess off‐target distribution (n = 3). (H) qPCR detection of AAV genome copy number in major organs (liver, heart, spleen, lung, kidney) (n = 3). (I–K) CLSM images of mouse liver sections from each treatment group 12 h later, showing AAV carrier localization (Cy5‐labeled, yellow), p21 (red), and cell nuclei (DAPI, blue) (n = 3). Scale bar: 20 µm. Data are presented as mean ± SD. ^*^ *p* < 0.05, ^**^ *p* < 0.01, ^***^ *p* < 0.001, and ^****^ *p* < 0.0001.

AAV (G1), KEV@AAV (G2), and SKEV@AAV (G3), all Cy5‐labeled, were then intravenously injected, and in vivo IVIS dynamic monitoring was conducted (Figure 4C). Quantitative analysis showed that SKEV@AAV fluorescence intensity in the p21‐enriched area was consistently higher than in the other two groups, starting at 6 h and peaking at 12 h, indicating preferential accumulation in lesions with high p21 expression (Figure 4D).

Ex vivo imaging at 48 h further confirmed this targeting. The fluorescence intensity of SKEV@AAV in tumor thrombus tissue was 4.37 and 1.86 times greater than that of G1 and G2, respectively (*p* < 0.0005), while signals in organs, such as the heart, spleen, lung, and kidney, were close to background levels (Figure 4E–G). qPCR analysis also revealed a significant increase in AAV genome copy number in tumor thrombus tissue with high *p21* expression (Figure 4H). CLSM further validated the colocalization of the Cy5^+^ signal from SKEV@AAV with the nuclei of p21‐positive cells (Figure 4I–K).

### 2.5 Verification of the In Vivo Therapeutic Efficacy of Senescence Reversal Strategies in an Orthotopic PVTT Model

Based on the verification of targeting specificity, an orthotopic PVTT model in nude mice was established using CSQT‐2‐Luc cells (Figure 5A) to systematically assess the potential of SKEV@AAV in regulating innate immunity [25]. Mice were intravenously administered PBS (G1), AAV (G2), KEV@AAV (G3), and SKEV@AAV (G4) on days 3, 6, 9, and 12 after tumor inoculation, with tumor progression monitored by bioluminescence imaging. In vivo imaging revealed that SKEV@AAV treatment produced a sustained antitumor effect starting from day 7, showing a 10.31‐fold reduction in tumor fluorescence intensity compared to the PBS group by day 14 (Figure 5B,C). Ex vivo IVIS imaging of the liver further confirmed that the fluorescence signal of tumor thrombi in the SKEV@AAV group was only 3.1% of that in the G1 group (*p* < 0.0001) and 6.7 times lower than that in the KEV@AAV group (p = 0.0077) (Figure 5D,E). Additionally, liver weight in the SKEV@AAV group was reduced by 2.31 times compared to the PBS group (*p* < 0.0001, Figure 5F), with no significant weight fluctuations observed throughout the study (Figure S47), indicating good biosafety. Given that nude mice lack T/B cells but retain a complete innate immune system, the regulatory effects of SKEV@AAV on sKCs, a key component of the innate immune system, were further investigated [26, 27]. qPCR analysis revealed that the expression of *p21* mRNA in liver tissues of the SKEV@AAV group was downregulated by 89.7% compared to the PBS group (*p* < 0.0001, Figure 5G). ELISA results indicated that the secretion levels of SASP core factors IL‐1α, IL‐1β, TGF‐β, and IL‐6 were reduced by 81.5%, 80.2%, 76.7%, and 78.0%, respectively (all *p* < 0.0001, Figure 5H).

**FIGURE 5:**
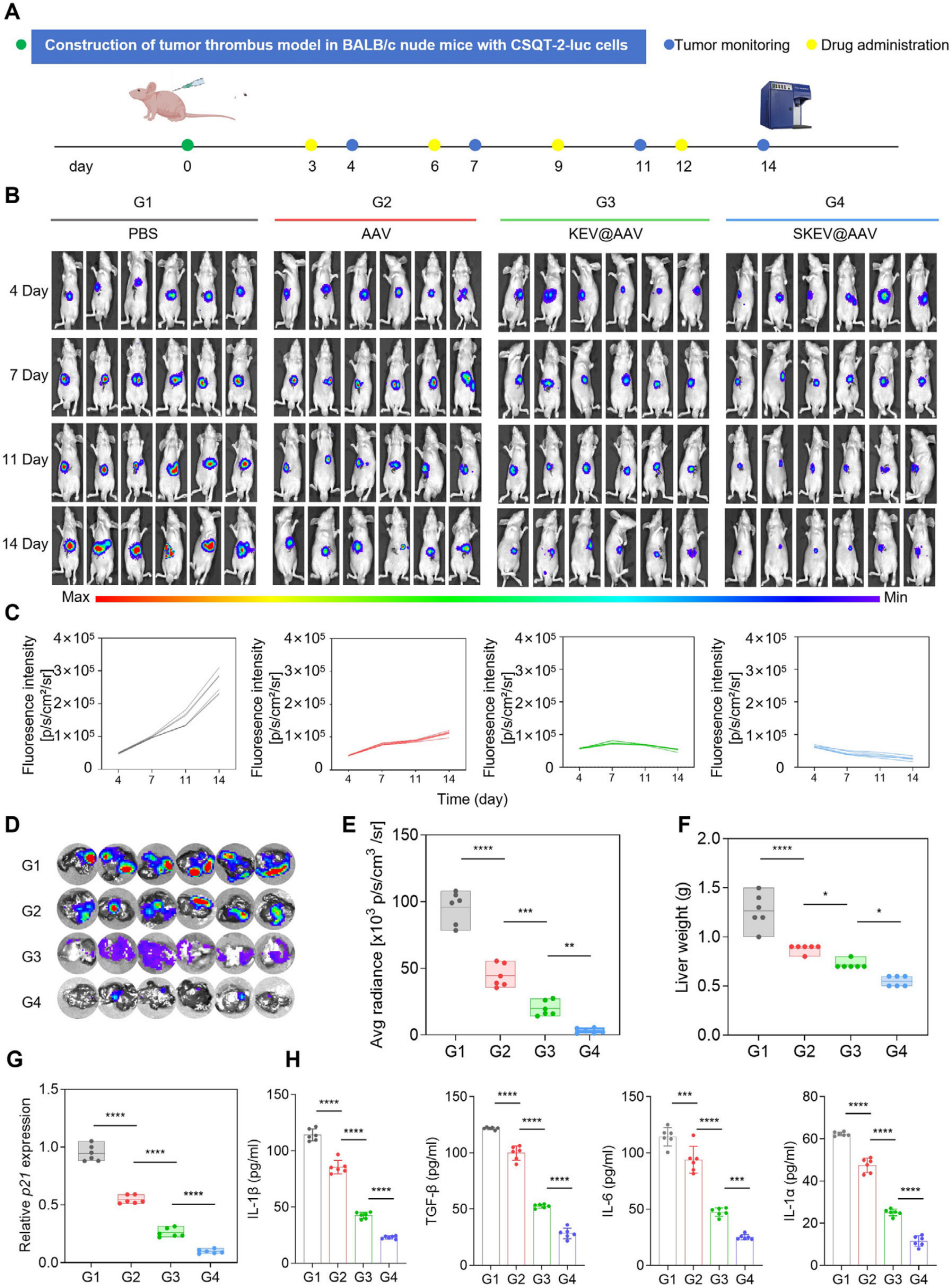
Antitumor effects of senescence reversal strategies in the orthotopic PVTT model. (A) Timeline of CSQT‐2 tumor inoculation (5 × 10^5^ cells per mouse) and treatment in the nude mouse model: tumor inoculation on day 0, bioluminescence imaging on day 3, and intravenous injections on days 3, 6, 9, and 12. (B) In vivo bioluminescence imaging on days 4, 7, 11, and 14 after tumor inoculation. Mice were treated with PBS (G1), AAV (G2), KEV@AAV (G3), or SKEV@AAV (G4). The color scale represents bioluminescence intensity (red: max; blue: min). (C) Quantitative analysis of in vivo bioluminescence intensity over time (n = 6). (D) Ex vivo IVIS imaging on day 14 showing the fluorescence intensity of tumor thrombi in liver tissues from each treatment group. (E) Quantitative analysis of fluorescence intensity of tumor thrombi in ex vivo liver tissues from each treatment group (n = 6). (F) Weight of ex vivo liver tissues from each treatment group on day 14 (n = 6). (G) qPCR analysis of *p21* expression in treated groups after treatment (n = 6). (H) ELISA detection of IL‐1α, IL‐1β, TGF‐β, and IL‐6 expression levels in mouse liver tissues from each treatment group after treatment (n = 6). Data are presented as mean ± SD. ^*^ *p* < 0.05, ^**^ *p* < 0.01, ^***^ *p* < 0.001, and ^****^ *p* < 0.0001.

Then, we performed immunofluorescence analysis on liver sections from PVTT‐bearing mice treated with PBS, AAV, KEV@AAV, or SKEV@AAV. The sections were co‐stained for CD68 and p21, with DAPI used to label nuclei. CD68^+^p21^+^ cells were readily detectable in tissues in the PBS and AAV groups, confirming the presence of p21‐high cells within these lesions. Compared with PBS, AAV, and KEV@AAV treatment, SKEV@AAV markedly reduced p21 signals in CD68^+^ cells in liver tissues. These findings provide direct histological evidence that SKEV@AAV decreases p21 expression within the CD68^+^ target‐cell compartment in vivo (Figure S48).

### 2.6 SKEV@AAV Combined with PD‐1 Blockade Significantly Inhibits the Growth of Splenic Metastatic HCC and Prolongs Survival

Building on the previously established ability of SKEV@AAV to reverse cellular senescence, its potential for combination therapy with PD‐1 immune checkpoint blockade in HCC treatment was further explored. The “in situ rejuvenation” of the tumor immune microenvironment by SKEV@AAV could synergize with PD‐1 inhibition to overcome immune suppression and activate a potent and durable antitumor immune response [28]. A splenic metastatic HCC model was first constructed, and significantly higher p21 accumulation in the HCC microenvironment compared to normal liver tissue was confirmed (Figures S49 and S50), along with the targeting properties of the three formulations: AAV, KEV@AAV, and SKEV@AAV (Figures S51 and S52). Hepa53.4‐Luc cells carrying the luciferase reporter gene were then used to construct the splenic metastatic HCC model. The corresponding treatments were administered via tail vein and intraperitoneal injection from day 4 to day 14, and tumor burden was monitored through in vivo bioluminescence imaging from day 3 to day 12 (Figure 6A), with survival tracked up to day 40. The results indicated that the tumor signal in the SKEV@AAV (G3) monotherapy group was reduced by 1.39 times compared to the KEV@AAV (G2) group, confirming that the senescence‐reversing EV formulation itself exhibits an antitumor effect (Figure 6B,C). In the combination therapy group, the fluorescence signal in the PD‐1 + SKEV@AAV combination group (G6) decreased by 222.84 times compared to the PD‐1 monoclonal antibody group (G4), with no significant change in mouse body weight (Figure S53). These data support an enhanced therapeutic effect of anti‐PD‐1 when combined with *p21*‐shRNA‐loaded exosomal formulations, with SKEV@AAV showing the strongest overall activity.

**FIGURE 6:**
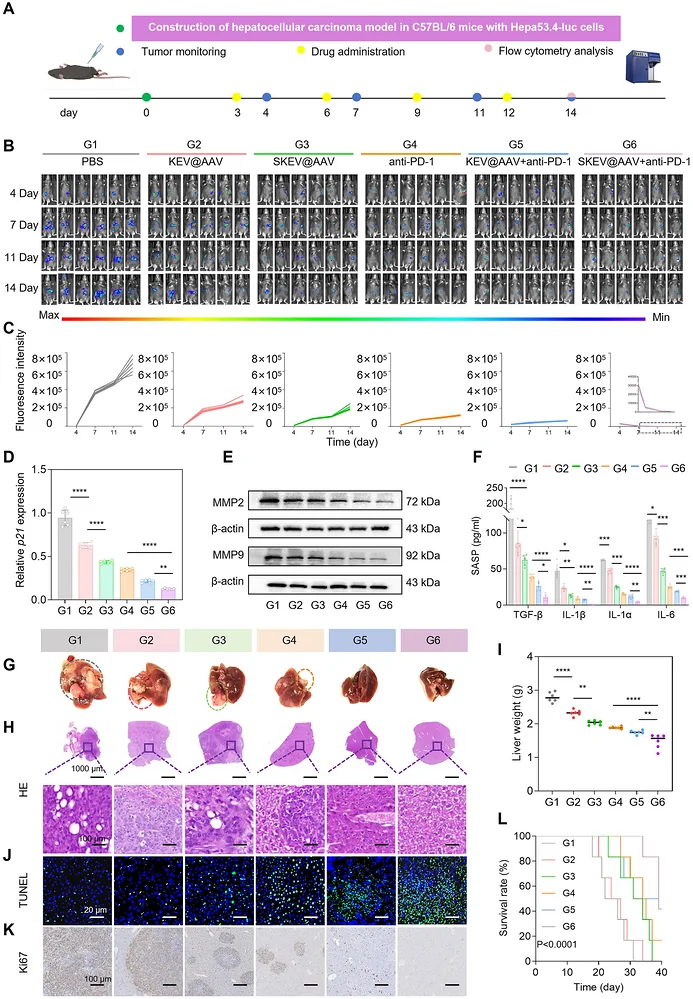
In vivo anti‐tumor efficacy of SKEV@AAV combined with PD‐1 blockade in a splenic‐metastatic HCC model. (A) Timeline of experiments after establishing the splenic‐metastatic HCC model with Hepa53.4‐Luc cells: tumor inoculation on day 0; tail‐vein and intraperitoneal injections on days 4, 7, 11, and 14; bioluminescence imaging on days 3, 6, 9, and 12; survival follow‐up until day 40. (B) Individual tumor growth curves and mean tumor growth kinetics for each group (n = 6). Groups: G1, PBS; G2, KEV@AAV; G3, SKEV@AAV; G4, anti‐PD‐1 antibody; G5, anti‐PD‐1 + KEV@AAV; G6, anti‐PD‐1 + SKEV@AAV. (C) Quantification of bioluminescence intensity of metastatic foci in each group (n = 6). (D) qPCR analysis of *p21* expression in treated groups (n = 6). (E) Western blot detection of MMP2 and MMP9 protein levels in treated groups (n = 3). (F) ELISA measurement of SASP factors (IL‐1α, IL‐6, IL‐1β, TGF‐β) in liver tissue lysates (n = 6). (G,H) Images of liver metastases and corresponding H&E staining; dashed lines indicate metastatic sites. (I) Ex vivo liver weights for each treatment group (n = 6). (J–K) TUNEL assay for apoptosis (scale bar: 20 µm) and Ki67 staining for proliferation (scale bar: 100 µm) in tumor tissue (n = 3). (L) Kaplan–Meier survival curves of mice in different treatment groups (n = 6). Data are presented as mean ± SD. ^*^ *p* < 0.05, ^**^ *p* < 0.01, ^***^ *p* < 0.001, and ^****^ *p* < 0.0001.

Mechanistically, anti‐PD‐1‐containing regimens were associated with reduced bulk *p21* levels (Figure 6D), decreased MMP2/MMP9 expression (Figure 6E; Figure S54), and lower concentrations of SASP‐associated cytokines in liver lesions (Figure 6F). Importantly, the decrease in *p21* should be interpreted cautiously. Studies have shown that PD‐L1‐positive senescent cells evade T cell‐mediated clearance, whereas PD‐1 blockade restores CD8^+^ T cell‐dependent senescence surveillance and reduces senescent cell burden in vivo [29, 30]. In our experiment, *p21* was measured in bulk liver‐lesion tissues. Therefore, reduced *p21* may reflect decreased tumor burden, altered macrophage composition, and reduced abundance of *p21*‐high senescent or senescence‐like immunosuppressive cells. Therefore, in our model, anti‐PD‐1 may lower tissue‐level *p21* by restoring immune‐mediated clearance of *p21*‐high senescent/immunosuppressive cells, rather than by directly suppressing *p21* expression.

In addition, the strong efficacy of KEV@AAV + anti‐PD‐1 can be explained by the fact that KEV@AAV is not an inert nanoparticle control, but rather an active comparator with the same gene‐silencing module and a different vesicle origin, because KEV@AAV and SKEV@AAV contained the same AAV‐*p21* shRNA payload. Therefore, KEV@AAV can attenuate the *p21*/SASP‐associated macrophage phenotype. This modulation may create a more permissive hepatic immune microenvironment for anti‐PD‐1‐mediated CD8^+^ T‐cell reactivation. This design allowed us to distinguish the contribution of the shared *p21*‐shRNA payload from the additional delivery advantage conferred by sKC‐derived vesicles.

Immunofluorescence staining of liver sections showed that SKEV@AAV treatment significantly reduced p21 signals in CD68^+^ cells compared with PBS, AAV, and KEV@AAV treatment. These findings provide direct histological evidence that SKEV@AAV decreases p21 expression within the CD68^+^ target‐cell compartment in vivo (Figure S55).

Ex vivo gross examination of the liver (Figure 6G; Figure S56) and H&E staining (Figure 6H) revealed that group G1 exhibited numerous diffuse metastatic nodules on the liver lobe surface, whereas no obvious tumor cell clusters were observed in group G6. Liver weight measurement showed that the average liver weight in group G6 was reduced by 47.2% compared to group G1 (*p* < 0.0001) (Figure 6I). TUNEL staining indicated an apoptosis rate of 65.58% in group G6, significantly higher than the 33.91% observed in group G1 (Figure 6J; Figure S57). Ki67 staining revealed a marked decrease in the proliferation index in group G6, further confirming that the combination therapy significantly induced apoptosis and inhibited cell proliferation (Figure 6K). Kaplan‐Meier survival analysis demonstrated that the median survival time in group G6 was extended to 40 days, significantly longer than the 24 days in group G1 (Figure 6L).

### 2.7 SKEV@AAV Combined with PD‐1 Reshapes the Immunosuppressive Liver Microenvironment and Elicits Potent Antitumor Immunity

To comprehensively evaluate the immunomodulatory effect of the combination therapy, the cytokine profile and immune‐cell populations within the treated hepatic tumors were systematically analyzed. ELISA of liver tissue lysates revealed that group G6 induced a pronounced shift toward a pro‐inflammatory, immunostimulatory phenotype. M1‐macrophage‐associated effectors, including inducible nitric oxide synthase (iNOS) and interferon‐β (IFN‐β), were significantly upregulated (Figure 7A,B), while immunosuppressive factors such as arginase‐1 (Arg‐1) and interleukin‐10 (IL‐10) were notably downregulated (Figure 7C,D).

**FIGURE 7:**
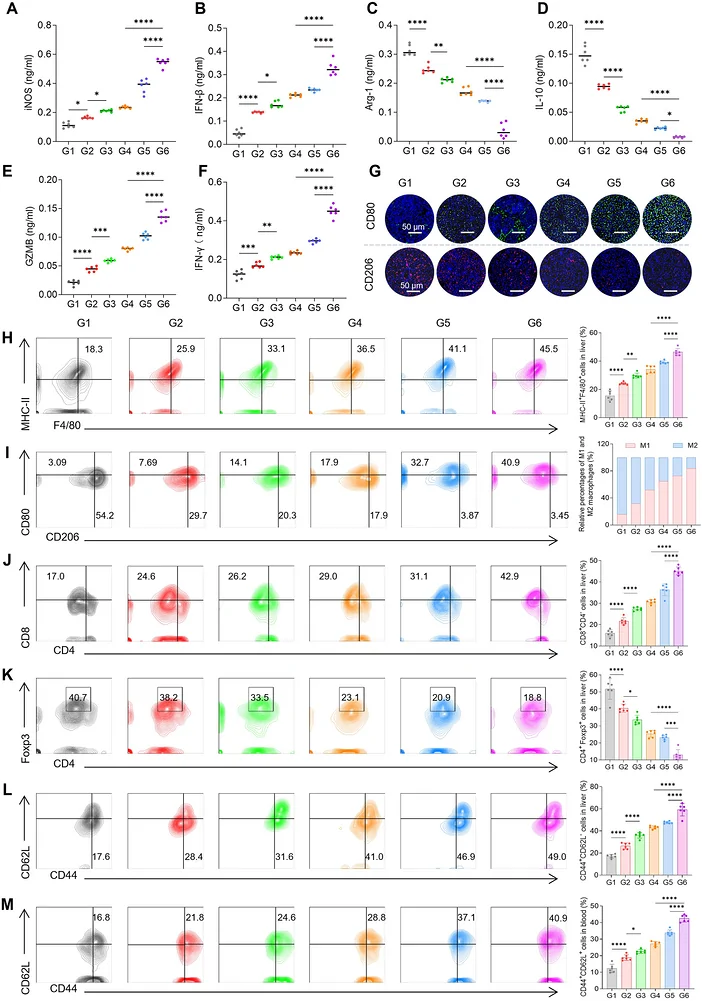
Immune‐activating capacity of SKEV@AAV combined with PD‐1 blockade. ELISA quantification of iNOS (A), IFN‐β (B), Arg‐1 (C), IL‐10 (D), GZMB (E), and IFN‐γ (F) in liver tissue lysates from each treatment group (n = 6). (G) Immunofluorescence images of CD80^+^ and CD206^+^ cells in liver tumor sections (n = 3): nuclei (DAPI, blue), CD80^+^ cells (green), CD206^+^ cells (red); scale bar: 50 µm. (H) Flow cytometry plots and relative quantification of MHC‐II^+^F4/80^+^ cells in mouse liver tissue (n = 6). (I) Flow cytometry plots and relative percentages of CD80 ^+^ F4/80^+^ and CD206^+^F4/80^+^ cells in mouse liver tissue (n = 6). (J) Flow cytometry analysis of CD8^+^CD3^+^ cells in mouse liver tissue (n = 6). (K) Flow cytometry plots and relative quantification of Treg cells (CD4^+^Foxp3^+^) in mouse liver tissue (n = 6). (L) Flow cytometry plots and relative quantification of effector‐memory T cells (CD62L^−^CD44^+^) in mouse liver tissue (n = 6). (M) Flow cytometry plots and relative quantification of central‐memory T cells (CD62L^+^CD44^+^) in peripheral blood (n = 6). Data are presented as mean ± SD. ^*^ *p* < 0.05, ^**^ *p* < 0.01, ^***^ *p* < 0.001, and ^****^ *p* < 0.0001.

Additionally, the combination therapy significantly increased the release of the cytotoxic T‐cell granule component granzyme B (GZMB) and the key effector cytokine interferon‐γ (IFN‐γ) (Figure 7E,F), indicating robust activation of CD8^+^ T‐cell‐mediated cytotoxicity. This cytokine shift was further confirmed by in vivo visualization and quantification of immune‐cell polarization and activation. Immunofluorescence staining showed that, compared to the monotherapy and control groups, liver sections from group G6 had significantly more CD80^+^ M1‐like macrophages and fewer CD206^+^ M2‐like macrophages (Figure 7G).

Flow cytometric analysis validated that SKEV@AAV plus anti‐PD‐1 induced the highest proportion of antigen‐presenting MHC‐II^+^ macrophages (Figure 7H; Figure S58a). This dual therapy most effectively polarized tumor‐associated macrophages (TAMs) toward the immunostimulatory M1 phenotype, as evidenced by the highest frequency of CD80^+^F4/80^+^ cells and the lowest frequency of CD206^+^F4/80 ^+^ cells (Figure 7I; Figure S58b).

In addition to innate activation, the impact of the combination therapy on adaptive immunity was assessed, particularly T‐cell compartments. Flow cytometry showed that SKEV@AAV plus anti‐PD‐1 significantly expanded CD8^+^CD3^+^ cytotoxic T cells within the liver tumors (Figure 7J; Figure S59a). Conversely, the proportion of immunosuppressive Tregs was most prominently decreased in group G6 (Figure 7K; Figure S59b), suggesting the dismantling of the immunosuppressive barrier.

The combination preferentially enriched effector‐memory T cells within the liver (Figure 7L; Figure S60a), while central‐memory T cells were enriched in the peripheral blood (Figure 7M; Figure S60b). This shift toward memory T‐cell subsets indicates that the treatment not only triggered a potent primary immune response but also likely established long‐term tumor‐specific immunologic memory, crucial for preventing relapse.

The combined use of SKEV@AAV and anti‐PD‐1 antibody reshapes the liver's immunosuppressive microenvironment at multiple levels. It enhances innate immune surveillance by driving macrophage polarization toward the M1 phenotype and boosting antigen presentation, while simultaneously unleashing adaptive immune potential through increased infiltration and function of cytotoxic CD8^+^ T cells, depletion of Tregs, and generation of memory T cells. This coordinated activation of innate and adaptive immunity underpins the superior antitumor efficacy observed with the combination regimen.

Finally, treatment safety was systematically evaluated through H&E staining of major organs (heart, spleen, kidney, lung) (Figure S61) and serum biochemical analyses (Figure S62). Results revealed intact tissue architecture in all groups, with no signs of inflammatory infiltration, necrosis, or other pathological changes. Key biochemical indices, including alanine aminotransferase (ALT), aspartate aminotransferase (AST), blood urea nitrogen (BUN), and creatinine (CREA), remained within normal ranges, further confirming the absence of systemic toxicity for this combination strategy.

## 3 Discussion and Conclusions

In recent years, aging research has made significant progress, particularly in the expansion and classification of aging biomarkers, the application of multi‐omics technologies, and deeper insights into the aging‐disease relationship [31, 32, 33]. By integrating single‐cell transcriptome sequencing, clinical sample validation, and nano‐drug‐delivery system development, this study systematically explored the pivotal role of *p21‐*high sKCs in the TME of HCC, particularly in patients with PVTT. The homing‐targeted delivery system SKEV@AAV, derived from sKCs' exosomes, efficiently silences *p21*, reverses the senescent phenotypes, and reshapes the immunosuppressive TME [34, 35]. These findings reveal, at both molecular and cellular levels, how sKCs orchestrate immune evasion and matrix remodeling through the SASP, thereby offering a potential innovative therapeutic strategy for advanced HCC, especially in the high‐risk PVTT subgroup.

Clinical specimens and single‐cell transcriptomes demonstrate a marked enrichment of sKCs in PT and PVTT tissues. The active intercellular crosstalk between sKCs, tumor cells, and fibroblasts suggests that sKCs act as key TME regulators, promoting tumor progression via paracrine mechanisms (Figures S17–S22). Notably, the increasing proportion of sKCs in PVTT correlates with disease severity, highlighting their potential as a biomarker for poor prognosis (Figure 1K). This aligns with recent findings showing that senescent macrophages in KRAS‐driven lung cancer promote tumor growth [12], suggesting that senescent immune cells may play a pivotal role in the TME of various solid tumors.

The successful construction of the SKEV@AAV nano‐system marks another breakthrough. By using exosomes as delivery vehicles, this system utilizes homologous targeting to precisely deliver *p21*‐shRNA. Proteomic profiling reveals significant enrichment of transmembrane transport and cell‐adhesion proteins in SKEV (Figure 2F–I), providing molecular insights into its homing specificity. In vitro and in vivo experiments consistently show that SKEV@AAV downregulates *p21*, suppresses SASP factor secretion, restores antigen presentation and endocytosis, and reduces the pro‐invasive and pro‐proliferative effects of sKCs on tumor cells and cancer‐associated fibroblasts (Figure 3G–J,M–O,T).

We next evaluated the therapeutic potential of SKEV@AAV in combination with immune checkpoint blockade. However, because the PVTT model was established in nude mice, it was used to evaluate single‐agent antitumor activity and innate immune remodeling, but not adaptive immune checkpoint combination responses (Figures 4, 5, 6). An immunocompetent splenic liver‐metastasis model was therefore used for evaluation of anti‐PD‐1 combination therapy. This model choice is biologically supported by recent spatial multi‐omics evidence in HCC‐PVTT showing that PVTT progression is not driven by malignant cells alone, but is critically shaped by a liver immune–stromal barrier [36, 37]. Therefore, although the splenic liver‐metastasis model does not fully recapitulate the intravascular architecture of PVTT, it preserves a liver‐colonizing immune–stromal microenvironment in which macrophage–fibroblast crosstalk, ECM remodeling, and T‐cell exclusion/reactivation are central determinants of therapeutic response. This provides a mechanistic rationale for using this immunocompetent model to test whether SKEV@AAV‐mediated reversal of *p21*‐high sKCs can potentiate PD‐1 blockade, while direct validation in an immunocompetent orthotopic PVTT model remains necessary (Figure 7; Figures S43–S45).

This study nonetheless has several important limitations. First, despite clear documentation of untreated status, cancer subtype in the scRNA‐seq cohort, the limited sample size and clinical granularity, particularly regarding broader biological context and clinical history, may still influence the sKC signature. Therefore, it still remains to be validated in larger‐scale studies with more patients and more comprehensive information.

Second, the lowered bulk‐tissue *p21* upon anti‐PD‐1 monotherapy is interpreted as a consequence of restored CD8^+^ T‐cell immune surveillance and clearance of *p21*‐high senescent/immunosuppressive cells, accompanied by reduced tumor burden and altered macrophage composition, rather than direct transcriptional repression of *p21*. However, we cannot definitively dissect the relative contribution of senescent cell clearance, tumor regression, and macrophage compositional shifts to the overall bulk *p21* reduction, and specific lineage‐tracing or cell‐depletion experiments are still required to further validate this mechanistic model.

Third, although short‐term safety appeared favorable, translational development of the AAV/exosome platform will require substantially deeper evaluation of long‐term immunogenicity, chronic toxicity, biodistribution persistence, off‐target transduction, and manufacturing reproducibility.

Future research should focus on elucidating the initiation and maintenance mechanisms of sKCs senescence, while optimizing scalable vector production and stability. The application of this strategy should be expanded to fibrotic diseases and other solid tumors, with translational research advancing, particularly for advanced HCC patients with PVTT. Clinical‐grade pre‐clinical and clinical trials of SKEV@AAV must be actively pursued, with rigorous evaluation of safety and efficacy in late‐stage HCC patients with PVTT, to offer new therapeutic hope for this currently untreatable population.

## 4 Materials and Methods

### 4.1 Ethics statement

All animal experimental protocols were performed in strict accordance with the Guidelines for the Care and Use of Laboratory Animals and were formally approved by the Laboratory Animal Ethics Committee of China Medical University (Protocol Number: CMUKT20251163). All human sample‐related experiments in this study were approved by the Ethics Committee of the First Hospital of China Medical University (Protocol Number: [2025] 422). All participants provided written informed consent to donate their biological samples.

### 4.2 Animals

Male C57BL/6 mice (8‐10 weeks old, RRID: MGI:2159769) and BALB/c nude mice (1‐5 weeks old, RRID: MGI:2161072) were used in this study. Humane endpoints included: tumor burden exceeding 10% of normal body weight, weight loss exceeding 20% of normal body weight, ulceration at the tumor site, or persistent self‐mutilation. These endpoints were approved by the Certification and Accreditation Administration of China (CNCA). Euthanasia was carried out by cervical dislocation under deep anesthesia.

### 4.3 Cell lines

ImKC cells (RRID: CVCL_HF55) were purchased from the Henan Industrial Microbial Strain Engineering Technology Research Center. NIH/3T3 cells (RRID: CVCL_0594) were obtained from Saibei Kang Medical Devices Co., Ltd. Hepa53.4‐Luc (luciferase‐labeled Hepa53.4), Hepa1‐6 (RRID: CVCL_0327), HuH7 (RRID: CVCL_0336), THP‐1 (RRID: CVCL_0006), LX‐2 (RRID: CVCL_5792), and CSQT‐2‐Luc (luciferase‐labeled CSQT‐2) cell lines were a gift from China Medical University. ImKC and THP‐1 cells were maintained in RPMI‐1640 supplemented with 10% fetal bovine serum (FBS) (Meilunbio, cat# PWL218), 100 U/mL penicillin, and 100 µg/mL streptomycin at 37°C in 5% CO_2_. THP‐1 monocytes were cultured and differentiated into macrophages using the modified phorbol 12‐myristate‐13‐acetate (PMA) technique. NIH/3T3, Hepa53.4 (RRID: CVCL_5765), Hepa53.4‐Luc, Hepa1‐6, HuH7, LX‐2, CSQT‐2 (RRID: CVCL_M183), and CSQT‐2‐Luc cells were cultured in Dulbecco's Modified Eagle Medium (DMEM) (Corning, cat# 10‐013‐CVR) with 10% FBS. Hepa53.4‐Luc and CSQT‐2‐Luc cells were transduced with lentivirus carrying the luciferase gene for bioluminescence imaging.

### 4.4 PT and PVTT Clinical Specimens

Resected PT and PVTT tissues were fixed, paraffin‐embedded, and serially sectioned for H&E staining to evaluate overall pathological changes. Subsequently, CLSM was used to visualize and semi‐quantify the immunofluorescence signals of p21 (RRID: AB_2838386) and CD68 (RRID: AB_2813855), assessing their expression intensity and cellular spatial localization.

### 4.5 Single‐Cell Transcriptome Sequencing

The following standardized workflow was used: 1) Patient Recruitment and Sample Collection: The study was approved by the Ethics Committee of the First Affiliated Hospital of China Medical University, and all enrolled patients diagnosed with HCC provided written informed consent and had not received chemotherapy, radiotherapy, or any other anti‐tumor drug treatments prior to sample collection. 2) Cell Preparation: Fresh tissues were washed with ice‐cold PBS (Hyclone SH30256.01) and mechanically‐enzymatically dissociated using the SeekGene SeekMate Tissue Dissociation Kit A Pro (K01801‐30). DNase I (Sigma cat# 9003‐98‐9) was added to reduce viscosity. Erythrocytes were lysed with Solarbio R1010. Viable cell number and viability were assessed with the SeekMate Tinitan Fluorescence Cell Counter (M002C) using AO/PI. Dead cells and debris were removed with Miltenyi 130‐109‐398/130‐090‐101 when necessary. Live cells were washed twice with RPMI‐1640 (Gibco 11875119) and resuspended in RPMI‐1640 containing 2% FBS (Gibco 10100147C) at 1 × 106 cells/mL. 3) Library Construction and Sequencing: The SeekOne DD Single Cell 3ʹ Library Kit (SeekGene K00202) was used for library preparation. A suitable number of cells was mixed with reverse‐transcription reagents and loaded onto a SeekOne chip S3. Barcoded Hydrogel Beads (BHBs) and partitioning oil were added to form emulsions. Reverse transcription was performed at 42°C for 90 min, then terminated at 85°C for 5 min. cDNA was emulsion‐broken, purified, PCR‐amplified, fragmented, end‐repaired, A‐tailed, adaptor‐ligated, and subjected to index PCR to enrich 3ʹ‐end sequences containing cell barcodes and UMIs. Libraries were purified using VAHTS DNA Clean Beads (Vazyme N411‐01), quality‐controlled with Qubit Q33226 and Bioptic Qsep400, and sequenced on the GeneMind SURFSeq 5000 platform in PE150 mode. 4) Data Pre‐processing and Quality Control: Seurat 5.1.0 was used for analysis. Cells with fewer than 200 UMIs, fewer than 200 genes, more than 7000 genes, or a mitochondrial transcript proportion greater than 10% were excluded [38]. A total of 71 915 high‐quality single‐cell transcriptomes from the public dataset (GSE149614) and 57 070 from the in‐house dataset were obtained. The top 2000 highly variable genes were used for principal component analysis (PCA); Harmony 1.2.0 was employed to remove batch effects, and SNN clustering was performed at resolution 0.5. 5) Differential Expression and Annotation: Seurat's FindAllMarkers function was used to generate cluster‐specific marker genes, while singleR, combined with manual marker genes and background knowledge, was used for cell type annotation. 6) Cell‐Cell Communication: CellChat 2.1.2 was used to quantitatively infer intercellular signaling networks, identify major incoming and outgoing signals for each cell type, and perform pairwise comparisons [39]. 7) Functional Module Scoring: Seurat's AddModuleScore was used to calculate senescence‐module scores for KCs clusters using the senescence‐related gene list provided in the Supporting Information. 8) Copy‐Number Variation: B cells (clusters 5 and 12) were used as a reference, and infercnv 1.20.0 detected gene‐level CNVs in intra‐sample hepatocytes (epithelial cells) [40]. 9) Statistics: Biological replicates were included for each scRNA‐seq model. Differential comparisons were made using unpaired Wilcoxon tests with FDR correction; the significance threshold was set at *p* < 0.05.

### 4.6 Construction and Characterization of *p21*‐shRNA‐AAV

miR30‐shRNA stem‐loop sequences targeting m*p21* and h*p21* were synthesized and cloned into the pAAV‐TBG‐Kozak‐EGFP‐[miR30‐shRNA]‐bGHpolyA backbone to generate the target plasmid. This plasmid was then mixed with the helper plasmid pHelper and the AAV8 serotype Rep/Cap plasmid pAAV‐RC in a 1:1:1 ratio and co‐transfected into HEK293T cells (RRID: CVCL_0063) using PEI (Polysciences, cat# 24765‐1). After 48–72 h, the culture supernatant and cells were collected, treated with Benzonase nuclease (Sigma, cat# E1014‐25KU), precipitated with PEG8000 (Sigma, cat# 89510–1KG‐F), and purified by a four‐step iodixanol density‐gradient ultracentrifugation. The 40%–60% interface virus band was collected, concentrated using 100 kDa ultra‐filters, buffer‐exchanged into PBS, 0.22 µm‐filtered, aliquoted, and titrated by qPCR for ITR genome copies. The final product was AAV8 carrying a liver‐specific TBG promoter, co‐expressing EGFP and *p21*‐shRNA [41, 42].

### 4.7 Induction of KCs Senescence and Exosome Isolation

3 × 10^5^ ImKC cells per well were seeded in 6‐well plates. After 24 h of adhesion, the medium was replaced with RPMI‐1640 containing 80 nmol/mL doxorubicin liposomes for 24 h, followed by 72 h of recovery in drug‐free medium [43]. Cells were washed twice with PBS to remove residual drug. The culture supernatant was sequentially centrifuged at 4°C: 300 × g for 10 min (live cells), 2000 × g for 10 min (dead cells), 10 000 × g for 30 min (organelles, apoptotic bodies, and membrane debris), and 100 000 × g for 1.5 h to pellet exosomes. The pellets were resuspended in 200 µL ice‐cold PBS. A BCA protein assay (cat# 23227) quantified KEV and SKEV protein content to assess yield and purity.

### 4.8 SA‐β‐Gal Senescence Assay

SA‐β‐gal staining (Beyotime, cat# C0602) was used to detect elevated β‐galactosidase expression in senescent cells [44]. Cells were grown to the log phase with typical morphology, fixed with 4% paraformaldehyde for 15 min, washed ≥3 times with PBS, incubated with SA‐β‐gal staining solution containing 5‐bromo‐4‐chloro‐3‐indolyl β‐D‐galactopyranoside at 37°C for 12–16 h, rinsed 1–2 times with PBS, and examined under a light microscope.

### 4.9 Preparation and Characterization of KEV@AAV and SKEV@AAV

The quality of the AAV preparation was first evaluated by negative‐staining TEM. Full and empty capsids were counted from representative images, showing 152 full capsids and 7 empty capsids, corresponding to 95.60% full particles. For each encapsulation reaction, 3 × 10^11^ AAV genome copies (GC) were used. According to the qPCR‐determined AAV titer of 1.0 × 10^13^ GC/mL, this corresponded to 30 µL of AAV stock. Based on nanoparticle tracking analysis, the exosome concentration was 4.5 × 10^10^ particles/mL, thus, 30 µL of exosome suspension contained approximately 1.35 × 10^9^ particles, yielding an input ratio of approximately 222 AAV GC per exosome particle.

The AAV–exosome mixture was sequentially extruded through 800, 400 nm, and 200 nm polycarbonate membranes for 10 cycles to obtain KEV@AAV or SKEV@AAV. After extrusion, the formulations were purified using a 30 kDa molecular‐weight‐cutoff centrifugal filter at 4000 rpm for 10 min to reduce residual free or unencapsulated components. The retained fraction was collected and stored at −80°C until use. Morphology was visualized by TEM. Size distribution was measured by DLS (Malvern). Exosomal markers CD81 (Absin, cat# abs113706, RRID: AB_10896976), CD63 (Abclonal, cat# A91512, RRID: AB_396631), and TSG101 (Absin, cat# Abs159883, RRID: AB_399937) were analyzed by Western blotting [45].

### 4.10 SDS‐PAGE

Equal amounts of protein from KCs, KEV, sKCs, and SKEV samples were mixed with 4 × Laemmli loading buffer (Meilunbio, cat# MA0003‐D_A), heated at 100°C for 10 min, and briefly centrifuged. Samples and a protein marker were loaded onto a precast 4%–20% SDS‐polyacrylamide gradient gel in a vertical tank with 1 × Tris‐glycine running buffer. Electrophoresis was performed at 120 V constant voltage for 60 min until the bromophenol blue front reached the gel bottom. The gel was stained with Coomassie Brilliant Blue R‐250 (Sigma, cat# 1125530025) for 30 min, destained until the background was clear, and imaged using a high‐resolution gel documentation system for qualitative protein profiling.

### 4.11 Cellular Uptake Study

Log‐phase KCs and sKCs were seeded at 5 × 10^4^ cells per well in 24‐well plates and allowed to adhere overnight at 37°C with 5% CO_2_. The medium was then replaced with serum‐free medium containing Dil‐fluorescently (Macklin, cat# D775877) labeled KEV@AAV or SKEV@AAV formulations, and incubation was continued for 2, 4, or 6 h. Afterward, cells were gently washed three times with ice‐cold PBS to remove unbound nanoparticles, fixed with 4% paraformaldehyde for 10 min, and stained with DAPI (10 µg/mL) for 10 min, followed by three additional PBS rinses. Subcellular localization of nanoparticles was visualized by CLSM, and MFI was quantified with ImageJ to calculate cellular uptake efficiency.

### 4.12 Proteomic Analysis

An appropriate volume of SDT lysis buffer (4% SDS, 100 mm Tris‐HCl, pH 7.6) was added to each sample for protein extraction, and protein concentration was determined by the BCA assay. For each sample, 15 µg of protein was mixed with 5 × loading buffer, boiled for 5 min, and separated by SDS‐PAGE (4%–20% pre‐cast gradient gel, 180 V constant, 45 min), followed by Coomassie Brilliant Blue R‐250 staining. Equal aliquots of all samples were pooled to generate a QC sample. All individual samples and the pooled QC were subjected to tryptic digestion using the filter‐aided proteome preparation (FASP) method. Peptides were desalted with C18 cartridges, lyophilized, and reconstituted in 40 µL of 0.1% formic acid, with peptide concentration measured at OD280. iRT standard peptides were spiked into each digest, and data‐independent acquisition (DIA) MS was performed on an Astral high‐resolution mass spectrometer.

DIA Analysis: Peptides were separated on a nano‐flow Vanquish Neo system (Thermo) and analyzed on an Astral mass spectrometer (Thermo Scientific). Positive‐ion mode; precursor scan range 380–980 m/z; MS1 resolution 240 000 at 200 m/z; normalized AGC target 500%; maximum injection time 5 ms. MS2 used DIA with 299 windows, a 2 m/z isolation window, HCD collision energy 25 eV, normalized AGC target 500%, and a maximum injection time of 3 ms. DIA raw files were processed with a search engine using the following parameters: enzyme trypsin, max missed cleavage 1, fixed modification carbamidomethyl (C), dynamic modifications oxidation (M), and acetyl (Protein N‐term); protein identifications were filtered at FDR < 1%.

### 4.13 In Vitro Encapsulation Efficiency and Stability

KEV@AAV and SKEV@AAV formulations were incubated in PBS (pH 7.4) containing 10% FBS at 37°C. Aliquots were collected on days 0, 1, 2, 3, 4, 5, 6, and 7, centrifuged, and lysed with 0.1% Triton X‐100 (Sigma, cat# T8787). Total DNA was quantified using NanoDrop, and viral genome copies in the lysate were determined by qPCR, comparing with the initial formulation to evaluate encapsulation efficiency and stability.

### 4.14 Cell Infection Assay

KCs and sKCs were seeded at 5 × 104 cells per well in 24‐well plates and cultured at 37°C, 5% CO_2_. GFP‐labeled KEV@AAV, SKEV@AAV, or naked AAV was diluted in serum‐free medium and used to replace the original medium. After 24 h of static incubation, the supernatant was removed, cells were gently washed twice with PBS, and complete medium containing 10% FBS was added for an additional 24 h. Cells were then fixed with 4% paraformaldehyde for 10 min, stained with DAPI (10 µg/mL) for 10 min, washed three times with PBS, and GFP signal was visualized by CLSM. MFI was quantified using ImageJ (RRID: SCR_003070) to calculate viral infection efficiency.

### 4.15 Effect of sKCs or sTHP‐1 Cells on the Proliferation of CSQT‐2, Hepa1‐6, and HuH7 Cells

CSQT‐2, Hepa1‐6, or HuH7 cells were seeded at 5 × 10^4^ cells per well in the lower chamber of 24‐well plates in complete DMEM. sKCs (5 × 10^4^ cells in 200 µL RPMI‐1640) or sTHP‐1 cells were placed in 0.4 µm Transwell inserts (Corning, cat# 3413) and co‐cultured at 37°C, 5% CO_2_. At 12, 24, and 48 h, lower‐chamber tumor cells were pulsed with 10 µm EdU (Meilunbio, cat# MA0425) for 2 h, fixed with 4% paraformaldehyde, and counterstained with Hoechst 33342. EdU‐positive tumor‐cell proliferation was quantified by confocal microscopy.

Subsequently, EV formulations were added to the upper‐chamber sKCs or sTHP‐1 cells for 24 h, followed by drug removal and a further 24h co‐culture in serum‐free medium. EdU staining was repeated to assess the proliferation of CSQT‐2, Hepa1‐6, or HuH7 cells. Supernatants were collected, and IL‐1α (Jymbio cat# JYM0009Mo), IL‐6 (Jymbio cat# JYM0012Mo‐T), IL‐1β (Jymbio cat# JYM0531Mo), and TGF‐β (Jymbio cat# JYM0144Mo) secretion levels were quantified by ELISA. RNA from sKCs was extracted using TRIzol, and *p21* expression was measured by qPCR to evaluate the ability of the formulations to downregulate *p21* in sKCs and thereby indirectly modulate tumor‐cell proliferation and SASP secretion.

### 4.16 Impact of sKCs or sTHP‐1 Cells on NIH/3T3 and LX‐2 Cells

CAFs were transformed from NIH/3T3 fibroblasts by supplementing 100 ng/mL recombinant mouse TGF‐β1 for 48 h. Next, NIH/3T3 or LX‐2 cells were seeded at 5 × 10^4^ cells per well in the lower chamber of 24‐well plates in complete DMEM. sKCs or sTHP‐1 cells  (5 × 10^4^ cells in 200 µL RPMI‐1640) were placed in 0.4 µm Transwell inserts and co‐cultured at 37°C, 5% CO_2_. After 24 h, MMP2 and MMP9 expression in lower‐chamber NIH/3T3 or LX‐2 cells was examined by Western blot and qPCR. Supernatants from the upper chamber were collected, and IL‐6 and TGF‐β levels were quantified by ELISA.

Subsequently, EV formulations were added to the upper‐chamber sKCs or sTHP‐1 cells for 24 h, followed by drug removal and a further 24h co‐culture in serum‐free medium. MMP2 (Absin, cat# abs119906) and MMP9 (Absin, cat# abs155182) expression was re‐examined by qPCR and Western blot, and IL‐6 and TGF‐β secretion was re‐quantified by ELISA to evaluate whether the formulations indirectly inhibit sKCs/sTHP‐1‐mediated effects on NIH/3T3 or LX‐2 cells via *p21* downregulation.

### 4.17 Flow‐Cytometric Analysis of Antigen‐Presentation Function

KCs and sKCs were centrifuged (300 × g, 5 min), washed twice with PBS, and stained with BV421‐anti‐MHC‐II (BD Biosciences, cat#562564, RRID:AB_2716857), PerCP‐Cy5.5‐anti‐F4/80 (BD Biosciences, cat#123128, RRID:AB_893484), APC‐Cy7‐anti‐CD80 (BD Biosciences, cat#553769, RRID:AB_395039), and FITC‐anti‐CD206 (BD Biosciences, cat# 141708, RRID:AB_10896057) for 30 min at 4°C. Flow cytometry was performed, and data were analyzed with FlowJo software to quantitatively evaluate the antigen‐presentation capacity of young versus senescent macrophages [46].

For treatment groups, sKCs were exposed to PBS, AAV, KEV@AAV, or SKEV@AAV for 24 h, then centrifuged (300 × g, 5 min), washed twice, stained with the same antibody cocktail for 30 min at 4°C, and analyzed by flow cytometry to quantify surface‐marker expression.

### 4.18 In Vitro Endocytosis Assay

KCs and sKCs were pre‐treated with cytochalasin D (7 µg/mL) for 2 h, then incubated with FITC‐dextran (1 mg/mL) for 6 h. After three washes, cells were fixed with 4% paraformaldehyde, rinsed four times with PBS, and mounted with DAPI‐containing Vectashield [47].

For drug‐treatment groups, FITC‐dextran (1 mg/mL) was added to sKCs together with PBS, AAV, KEV@AAV, or SKEV@AAV for 6 h. Samples were observed under a confocal microscope.

### 4.19 Bulk RNA‐Seq of CSQT‐2 Cells Treated with THP‐1 or sTHP‐1 Cells

Total RNA was extracted from CSQT‐2 cells. Briefly, the culture medium was discarded, and the cells were quickly washed with PBS. After removing PBS, the cells were directly lysed in their culture dish using Trizol by repeated pipetting until complete dissolution. The lysate was stored at −80°C for subsequent RNA isolation. Total RNA meeting quality control standards (RIN > 7) as determined by the Agilent 2100 Bioanalyzer was then used for library construction with the Hieff NGS MaxUp II Dual‐mode mRNA Library Prep Kit for Illumina. Briefly, mRNA containing poly(A) tails was enriched from total RNA using Oligo(dT) magnetic beads. The purified mRNA was then fragmented to a target size of approximately 300–350 bp in Frag/Prime Buffer at 94°C. Using the fragmented mRNA as a template, first‐strand cDNA was synthesized with random primers and the 1st Strand Enzyme Mix. This was followed by second‐strand cDNA synthesis using the dNTP‐containing 2nd Strand Buffer and 2nd Strand Enzyme Master Mix, during which the reaction simultaneously performed end repair and dA‐tailing to generate blunt‐end, dA‐tailed double‐stranded cDNA. Illumina sequencing adapters were then ligated to the cDNA ends using Quick T4 DNA Ligase. The ligation products were purified using Hieff NGS DNA Selection Beads, and the final library was amplified by PCR with the kit's 2 × Super Canace II High‐Fidelity Mix and Primer Mix to enrich for properly ligated fragments and incorporate index sequences. The resulting libraries were quality‐checked for size distribution using the Agilent 2100 Bioanalyzer and qualified libraries were sequenced on the Illumina NovaSeq X platform for PE150 reads.

### 4.20 In Vivo Biodistribution Study in an Orthotopic Tumor‐Thrombus Model

Male BALB/c nude mice underwent laparotomy, and 5 × 10^5^ CSQT‐2 cells (in 50 µL) were precisely inoculated into the left hepatic lobe to establish an orthotopic tumor‐thrombus model. Tumor‐bearing mice were randomly assigned to three groups (n = 6) and injected via the tail vein with AAV, KEV@AAV, or SKEV@AAV at an AAV dose of 1 × 10^12^ vg/kg. Near‐infrared signals were acquired at 1, 6, 12, 24, and 48 h post‐injection using an IVIS Lumina Series III in vivo imaging system (excitation 745 nm, emission 800 nm). At 48 h, the mice were euthanized, and the heart, liver, spleen, lung, and kidney were excised for ex vivo fluorescence imaging. Tissue DNA was extracted, and AAV genome copies in each organ were quantified by qPCR. Simultaneously, frozen liver sections were fixed with 4% paraformaldehyde, permeabilized, and stained with 10 µg/mL DAPI for 10 min. Accumulation of Cy5‐labeled (Meilunbio, cat# MB12195) formulations in the liver was visualized by CLSM.

### 4.21 Biodistribution Study in a Splenic Metastatic Liver‐Cancer Model

Laparotomy was performed, and 5 × 10^5^ Hepa53.4 cells (50 µL) were injected into the splenic parenchyma to establish a splenic metastatic liver cancer model. Tumor‐bearing mice were randomly divided into three groups (n = 6) and injected via the tail vein with AAV, KEV@AAV, or SKEV@AAV at an AAV dose of 1 × 10^12^ vg/kg. Near‐infrared signals were acquired at 1, 6, 12, 24, and 48 h post‐injection using an IVIS Lumina Series III in vivo imaging system.

### 4.22 In Vivo Antitumor Efficacy Evaluation

A PVTT model was established by open injection of 5 × 10^5^ CSQT‐2 cells (50 µL) into the left hepatic lobe of male BALB/c nude mice. Tumor‐bearing mice were randomly assigned to four groups (n = 6) and treated intravenously with PBS, AAV, KEV@AAV, or SKEV@AAV. AAV formulations were administered at a dose of 1 × 10^12^ vg/kg every 3 days. Body weight was recorded daily, and tumor fluorescence intensity was monitored on days 4, 7, 11, and 14 using the IVIS Lumina system to assess tumor progression dynamically. On day 14, mice were euthanized by cervical dislocation; livers were excised, weighed, and imaged for fluorescence to compare tumor inhibition among groups.

### 4.23 In Vivo Antitumor Efficacy of Formulations Combined with PD‐1 Blockade

C57BL/6 male mice (8–10 weeks old) underwent splenic injection of 5 × 10^5^ Hepa53.4 cells (50 µL) to establish a splenic metastatic liver cancer model. A splenic injection model was employed to establish PVTT‐bearing mice, as previously validated [48].

The animals were randomly assigned to six groups (n = 6) and received tail‐vein injections of PBS, KEV@AAV, or SKEV@AAV, or intraperitoneal injections of PD‐1 antibody (200 µg/mouse), along with two combination groups: PD‐1 + KEV@AAV and PD‐1 + SKEV@AAV. AAV formulations were administered at a dose of 1 × 10^12^ vg/kg every 3 days. Body weight was monitored daily, and tumor fluorescence was assessed twice weekly using the IVIS Lumina system. On day 14, the mice were euthanized, and livers were excised, weighed, and photographed to compare tumor inhibition. SASP factors were quantified by ELISA to evaluate modulation of the immune microenvironment. KCs were isolated by collagenase digestion, and *p21* expression was measured by qPCR. Liver tissues were fixed in 4% paraformaldehyde, paraffin‐embedded, and subjected to H&E staining. After antigen retrieval in pH 6.0 sodium citrate buffer at 95°C and blocking with 5% goat serum, sections were incubated with Ki67 (Affinity Biosciences, AF0198, RRID: AB_2834152) primary antibody at 4°C, followed by Alexa Fluor‐conjugated secondary antibody to assess proliferation. Apoptosis was detected by TUNEL staining. Images were acquired using CLSM and quantified.

### 4.24 In Vivo Antitumor Immune Response Analysis

Tumor tissues were harvested from Hepa53.4‐bearing C57BL/6 male mice (8‐10 weeks old). Orbital blood (200 µL) was collected into EDTA tubes. Lymphocytes were isolated by centrifugation according to kit instructions. Tumor tissues were minced in 2 mL of 1.5 mg/mL collagenase IV (Bioshap, cat# BS165) and digested at 37°C with gentle shaking for 1 h to obtain single‐cell suspensions. Then a mix of fluorochrome‐conjugated primary antibody, including APC‐CY7 anti‐CD45 (BD Biosciences, cat# 557659, RRID:AB_396774), PerCP anti‐F4/80 (BD Biosciences, cat# 123128, RRID:AB_893484), APC anti‐CD206 (BD Biosciences, cat# 141708, RRID:AB_10900231), and PE anti‐CD80 (BD Biosciences, cat# 553769, RRID:AB_395039), BV421 anti‐MHC‐II (BD Biosciences, cat# 562564, RRID:AB_2716857), FITC anti‐CD11b (BD Biosciences, cat# 553312, RRID:AB_398535), FITC anti‐CD3 (BD Biosciences, cat# 100204, RRID:AB_312661), APC anti‐CD62L (Biolegend, cat# 104412, RRID:AB_313099), PE anti‐CD44 (MyBioSource Cat# MBS532132, RRID:AB_10884730), Percp‐cy5.5 CD8a (BD Biosciences Cat# 551162, RRID:AB_394081), APC anti‐CD4 (BD Biosciences Cat# 553051, RRID:AB_398528), PE anti‐Foxp3 (BD Biosciences Cat# 560044, RRID:AB_1645589) and Percp‐cy5.5 anti‐CD3 (BD Biosciences Cat# 565983, RRID:AB_2739435), was added to cells and gently mixed. Flow cytometry was used to determine the proportion of stained cells in tumor tissues, and data were analyzed with FlowJo software [49].

After sorting, ELISA was performed to measure cytokines from macrophages [IFN‐β (Jymbio cat# JYM0332Mo), Arg‐1 (Jymbio cat# JYM0682Mo), IL‐10 (Jymbio cat# JYM0005Mo), iNOS (Jymbio cat# JYM0448Mo)] and CD8^+^ T cells [IFN‐γ (Jymbio cat# JYM0540Mo), GzmB (Jymbio cat# JYM0261Mo)]. Hepatic sections were subjected to CD80 (Abclonal, A26801, RRID: AB_1544668) / CD206 (Abclonal, A26948, RRID: AB_394065) immunofluorescence and confocal microscopy to quantify macrophage phenotypes. Survival and differences were analyzed by log‐rank test and ANOVA to comprehensively evaluate the antitumor effect of SKEV@AAV combined with PD‐1 blockade.

### 4.25 In Vivo Toxicity Evaluation

Body weight was monitored throughout the efficacy studies. At the end of the experiments, all mice were euthanized, and major organs (heart, liver, spleen, lung, and kidney) were collected for H&E staining. Whole blood was collected for hematological analysis, and serum was isolated for liver and kidney function tests.

### 4.26 Statistical Analysis

All statistical analyses were performed using GraphPad Prism 9.0. All quantitative data are presented as mean ± SEM. p‐values < 0.05 were considered statistically significant. A t‐test was used to determine the significance of differences between two groups. A one way analysis of variance (ANOVA) was used to compare multiple groups. Statistical significance was defined as *p* < 0.05, *p* < 0.01, *p* < 0.001, and *p* < 0.0001.

## Author Contributions

**Na Ta**: Data curation, validation, investigation, writing the original draft, writing – review, and editing; **Shuyi Wang**: Data curation, formal analysis, validation, writing – review, and editing; **Boyan Zhang**: Data curation and formal analysis; **Ning Liu**: Conceptualization, formal analysis, validation; **Yingchen Han**: Data curation and formal analysis; **Aoran Liu**: Data curation and formal analysis; **Ying Xu**: Data curation and formal analysis; **Tingsong Chen**: Conceptualization, formal analysis, validation, investigation, writing – review, and editing; **Ye Zhang**: Resources, writing – review and editing; **Qiuhua Luo**: Conceptualization, data curation, resources, formal analysis, supervision, writing, review, and editing; **Tao Han**: Conceptualization, resources, formal analysis, supervision, investigation, validation, funding acquisition, writing – review, and editing.

## Conflicts of Interest

The authors declare no conflicts of interest.

## Clinical Trial Registration

This study does not involve a clinical trial as defined by the International Committee of Medical Journal Editors (ICMJE) or the WHO, and therefore clinical trial registration was not required.

## Supporting information




**Supporting File 1**: advs76384‐sup‐0001‐SuppMat.docx.

**Supporting File 2**: advs76384‐sup‐0002‐Materials.doc.

## Data Availability

The data that support the findings of this study are available from the corresponding author upon reasonable request.
