# Supplementary material for: Targeting p21‐High Senescent Kupffer Cells Nanotherapeutically Potentiates Antitumor Immunity in Advanced Hepatocellular Carcinoma with Portal Vein Tumor Thrombus
Source: Adv Sci (Weinh). 2026 Jul 2;13(54):e76384. doi: 10.1002/advs.76384 (PMC13336571; doi:10.1002/advs.76384)
Supplement: Supplementary file 1 [file ADVS-13-e76384-s001.docx]

**Supplementary Materials for**

**Targeting *p21*-high senescent Kupffer cells nanotherapeutically potentiates antitumor immunity in advanced hepatocellular carcinoma with portal vein tumor thrombus**

*Na Ta1,2#, Shuyi Wang1#, Boyan Zhang3#, Ning Liu4#, Yingchen Han1, Aoran Liu5, Ying Xu6, Tingsong Chen7*, Ye Zhang5*, Qiuhua Luo3*, Tao Han1**

**Affiliations:**

1Department of Medical Oncology, The First Hospital of China Medical University, Shenyang, 110001, P. R. China.

2Department of Neurology, The Second Affiliated Hospital of Dalian Medical University, Dalian, 116027, P. R. China.

3Department of Pharmacy, The First Hospital of China Medical University, Shenyang, Liaoning, 110001, P. R. China.

4Department of Pancreatic and Biliary Surgery, The First Affiliated Hospital of China Medical University,Shenyang, Liaoning, 110001, P. R. China.

5The First Laboratory of Cancer Institute, The First Hospital of China Medical University, Shenyang Liaoning, 110001, P.R. China.

6School of Medicine, Southern University of Science and Technology, Shenzhen, 518055, China.

7Department of Interventional Oncology, Seventh People's Hospital of Shanghai University of Traditional Chinese Medicine, Shanghai, 200137, P. R. China.

\# These authors contributed equally to this work.

***Correspondence** E-mail: than1984@sina.com(T.H.); [qhluo@cmu.edu.cn(Q.](mailto:qhluo@cmu.edu.cn(QH)L.); [yzhang21@cmu.edu.cn(Y.Z.);](mailto:yzhang21@cmu.edu.cn(Y.Z.);) cts552052597@163.com(T.C.)

**Figure S1.** t-SNE plot of 71,915 cells, which are clustered into 18 groups.

**Figure S2.** Dot plot showing the percentage of cells expressing canonical marker genes and their average expression levels across 19 cell clusters.

a

c

b

**Figure S3. a.** t-SNE plot of 12,831 Mon/Mac. **b.** Dot plot showing the percentage of cells expressing canonical marker genes and their average expression levels.**c.** t-SNE plot of 11,746 KCs, clustered into 20 subsets.

a

b

**Figure S4.** **a.** t-SNE plot of 57,070 cells, clustered into 19 subsets and cell annotation. **b.** Dot plot showing the percentage of cells expressing canonical marker genes of major cell types and their average expression levels across 19 cell clusters in the self-generated dataset.

b

a

c

**Figure S5.** **a.** t-SNE plot of 12,831 Mon/Mac. **b.** Dot plot showing the percentage of cells expressing canonical marker genes and their average expression levels.**c.** t-SNE plot of 11,746 KCs, clustered into 20 subsets.

**Figure S6.** The Proportions of *p21*-high and *p21*-low KCs clusters across different groups.

**Figure S7.** Copy number variation landscape across hepatocyte clusters. Clusters 5 and 12 served as reference cells with stable genomes, while Clusters 8 and 10, identified as hepatocytes, exhibited severe genomic gains and losses and were defined as malignant.

**Figure S8.** H&E staining of normal human liver tissue. Scale bar: 50 μm.

**Figure S9.** Co-localization of CD68 and p21 in normal human liver tissue. **a.** Representative confocal microscopy images showing the expression of CD68 (green) and p21 (red) in normal human liver tissue (n = 3). Nuclei were counterstained with DAPI (blue). Scale bar: 20 μm. **b.** Quantitative analysis of CD68⁺ p21⁺ cells in normal human liver tissue.

**Figure S10.** **a.** Bright-field images and SA-β-Gal staining of KCs treated with increasing concentrations of doxorubicin (30–150 nmol/mL) (n = 3). Scale bar: 100 μm. **b.** Quantitative analysis of SA-β-Gal-positive cells under different doxorubicin concentrations.

**Figure S11.** Western blot analysis of KCs treated with increasing concentrations doxorubicin (30-150 nmol/mL), with β-actin as a loading control (n = 3). Data are presented as mean ± SD. **p* < 0.05, ***p* < 0.01, ****p* < 0.001, and *****p* < 0.0001.

**Figure S12. a**.CLSM imaging evaluating the proliferation of KCs and sKCs cells (Red, EdU staining; Blue, DAPI staining). Scale bar: 50 μm. **b.** Fluorescence intensity quantification of the proliferation status of KCs and sKCs (n = 3). Data are presented as mean ± SD. **p* < 0.05, ***p* < 0.01, ****p* < 0.001, and *****p* < 0.0001.

**Figure S13.** Quality control of AAV preparation for SKEV@AAV. TEM negative staining analysis showing full and empty AAV capsids. Full particles: 152; empty particles: 7; full/empty ratio: 95.60% full. Scale bar: 100 nm.

**Figure S14.** Western blot analysis of CD63, TSG101, and CD81 expression in AAV, KCs, sKCs, KEV, KEV@AAV, SKEV, and SKEV@AAV.

**Figure S15.** **a.** The encapsulation efficiency of KEV@AAV and SKEV@AAV was assessed by qPCR in vitro (n = 3). **b.** The stability of the formulations was evaluated by qPCR at different time points.

**Figure S16.** Co-localization of GFP-labeled AAV (green) with KEV-Dil (red, KEV@AAV) and SKEV-Dil (red, SKEV@AAV). Scale bar: 10 μm.

**Figure S17.** Zeta potential of AAV, KEV, KEV@AAV, SKEV, and SKEV@AAV measured by dynamic light scattering (n = 3).

**Figure S18.** Cytotoxicity of KEV@AAV and SKEV@AAV on RAW264.7, NIH/3T3, and DC2.4 cells. Cells were treated with PBS, AAV, KEV@AAV, or SKEV@AAV at therapeutic doses (n = 3). Data are presented as mean ± SD. **p* < 0.05, ***p* < 0.01, ****p* < 0.001, and *****p* < 0.0001.

**Figure S19.** Hepatic toxicity test of SKEV@AAV in healthy mice (H&E staining) (n = 3). Scale bar: 1000 μm (left), 100 μm (right).

**Figure S20. a.** KCs and sKCs were co-cultured and incubated with Dil-labeled SKEV@AAV (SKEV@AAV-Dil) and Dir-labeled KEV@AAV (KEV@AAV-Dir). Subsequently, flow cytometry was used to detect the uptake of SKEV@AAV-Dil by KCs and sKCs. **b.** The gating strategy for Dil⁺ cells analysis in vitro. **c.** Flow cytometry was used to detect the uptake of KEV@AAV-Dir by KCs and sKCs. **d.** The gating strategy for Dir⁺ cells analysis in vitro (n = 3). Data are presented as mean ± SD. **p* < 0.05, ***p* < 0.01, ****p* < 0.001, and *****p* < 0.0001.

.

**Figure S21.** **a.** Confocal microscopy images showing the effect of sTHP-1 cells co-cultured with HuH7 cells for 12 h, 24 h, and 48 h on HuH7 cells proliferation. Scale bar: 50 μm. **b.** Quantitative analysis of the percentage of EdU-positive HuH7 cells after co-culture with sTHP-1 cells at 12 h, 24 h, and 48 h (n = 3). Data are presented as mean ± SD. **p* < 0.05, ***p* < 0.01, ****p* < 0.001, and *****p* < 0.0001.

**Figure S22. a.** Confocal images of Hepa1-6 cells proliferation after treatment with sKCs pre-loaded with PBS (G1), AAV (G2), KEV@AAV (G3), or SKEV@AAV (G4). Scale bar: 100 μm. **b.** Quantitative analysis of the percentage of EdU-positive Hepa1-6 cells (n = 3). Data are presented as mean ± SD. **p* < 0.05, ***p* < 0.01, ****p* < 0.001, and *****p* < 0.0001.

**Figure S23.** **a.** Confocal images of HuH7 cells proliferation after treatment with sTHP-1 cells pre-loaded with PBS (G1), AAV (G2), TEV@AAV (G3), or STEV@AAV (G4). Scale bar: 100 μm. **b.** Quantification of proliferation fluorescence intensity (n = 3). Data are presented as mean ± SD. **p* < 0.05, ***p* < 0.01, ****p* < 0.001, and *****p* < 0.0001.

**Figure S24.** **a.** Confocal images of CSQT-2 cells proliferation after treatment with sTHP-1 cells pre-loaded with PBS (G1), AAV (G2), TEV@AAV (G3), or STEV@AAV (G4). Scale bar: 100 μm. **b.** Quantification of proliferation fluorescence intensity (n = 3). Data are presented as mean ± SD. **p* < 0.05, ***p* < 0.01, ****p* < 0.001, and *****p* < 0.0001.

**Figure S25. a.** Confocal images of CSQT-2 cells proliferation after treatment with PBS or STEV@AAV. Scale bar: 50 μm. **b.** Quantification of EdU-positive cells (n = 3). **c.** After the addition of PBS or STEV@AAV to the supernatant of CSQT-2 cells, qPCR showed the mRNA expression of *p21* in each treated group (n = 3). Data are presented as mean ± SD. **p* < 0.05, ***p* < 0.01, ****p* < 0.001, and *****p* < 0.0001.

**Figure S26.** **a.** Confocal images of Hepa1-6 cells proliferation after treatment with PBS or SKEV@AAV. Scale bar: 50 μm. **b.** Quantification of EdU-positive cells (n = 3). **c.** After the addition of PBS or SKEV@AAV to the supernatant of Hepa1-6 cells, qPCR showed the mRNA expression of *p21* in each treated group (n = 3). Data are presented as mean ± SD. **p* < 0.05, ***p* < 0.01, ****p* < 0.001, and *****p* < 0.0001.

**Figure S27.** ELISA quantification of SASP factors (IL-6, IL-1α, IL-1β, TGF-β) in sKCs’ supernatants at indicated time points (n = 3). Data are presented as mean ± SD. **p* < 0.05, ***p* < 0.01, ****p* < 0.001, and *****p* < 0.0001.

**Figure S28.** **a.** Confocal micrographs comparing the endocytosis in KCs and sKCs after cytochalasin D treatment (n = 3). Scale bar: 100 μm. **b.** Quantification of endocytic activity by MFI. Data are presented as mean ± SD. **p* < 0.05, ***p* < 0.01, ****p* < 0.001, and *****p* < 0.0001.

**Figure S29.** Flow cytometric analysis of MHC-II expression on the surface of KCs and sKCs (n = 3). Data are presented as mean ± SD. **p* < 0.05, ***p* < 0.01, ****p* < 0.001, and *****p* < 0.0001.

**Figure S30. a.** Flow cytometry analysis of CD8⁺ T cells infiltration in co-cultures of T cells with sKCs (n = 3)**. b.** The gating strategy for CD8⁺ T cells analysis in vitro. **c.** Flow cytometry analysis of Treg cells infiltration in co-cultures of T cells with sKCs (n = 3)**. d.** The gating strategy for Treg cells analysis in vitro. Data are presented as mean ± SD. **p* < 0.05, ***p* < 0.01, ****p* < 0.001, and *****p* < 0.0001.

**Figure S31.** **a.** Confocal microscopy assessment of endocytic activity in sKCs following different treatments. Treatment groups: PBS (G1), AAV (G2), KEV@AAV (G3), SKEV@AAV (G4). Scale bar: 100 μm. **b.** Quantitative analysis of endocytic activity, presented as MFI (n = 3). Data are presented as mean ± SD. **p* < 0.05, ***p* < 0.01, ****p* < 0.001, and *****p* < 0.0001.

**Figure S32. a.** Confocal images showing the proliferation of sKCs following treatment with PBS, SKEV, AAV, KEV@AAV, or SKEV@AAV. Scale bar: 50 μm. **b.** Quantification of EdU-positive sKCs following treatment with PBS, SKEV, AAV, KEV@AAV, or SKEV@AAV (n = 3). Data are presented as mean ± SD. **p* < 0.05, ***p* < 0.01, ****p* < 0.001, and *****p* < 0.0001.

**Figure S33.** qPCR analysis of *p21* gene expression in sKCs following treatment with PBS, AAV, SKEV, KEV@AAV, or SKEV@AAV. Relative expression normalized to PBS control (n = 3). Data are presented as mean ± SD. **p* < 0.05, ***p* < 0.01, ****p* < 0.001, and *****p* < 0.0001.

**Figure S34.** Quantitative analysis of MMP2 protein expression levels in NIH/3T3 cells by Western blotting was performed following co-culture with KCs and sKCs (n = 3). Data are presented as mean ± SD. **p* < 0.05, ***p* < 0.01, ****p* < 0.001, and *****p* < 0.0001.

**Figure S35.** Quantitative analysis of MMP9 protein expression levels in NIH/3T3 cells by Western blotting following co-culture with KCs and sKCs (n = 3). Data are presented as mean ± SD. **p* < 0.05, ***p* < 0.01, ****p* < 0.001, and *****p* < 0.0001.

**Figure S36.** **a.** Western blot analysis of MMP2 protein expression in LX-2 cells following co-culture with THP-1 cells and sTHP-1 cells. β-actin was used as a loading control. **b.** Quantitative analysis of MMP2 protein expression levels in LX-2 cells (n = 3). Data are presented as mean ± SD. **p* < 0.05, ***p* < 0.01, ****p* < 0.001, and *****p* < 0.0001.

**Figure S37.** **a.** Western blot analysis of MMP9 protein expression in LX-2 cells following co-culture with THP-1 cells and sTHP-1 cells. β-actin was used as a loading control. **b.** Quantitative analysis of MMP9 protein expression levels in LX-2 cells (n = 3). Data are presented as mean ± SD. **p* < 0.05, ***p* < 0.01, ****p* < 0.001, and *****p* < 0.0001.

**Figure S38.** qPCR analysis of *MMP2* and *MMP9* expression in LX-2 cells after transwell co-culture with THP-1 cells and sTHP-1 cells (n = 3). Data are presented as mean ± SD. **p* < 0.05, ***p* < 0.01, ****p* < 0.001, and *****p* < 0.0001.

**Figure S39.** Quantitative analysis of MMP2 protein expression levels in NIH/3T3 cells by Western blotting was performed following co-culture with sKCs. Treatment groups: PBS (G1), AAV (G2), KEV@AAV (G3), SKEV@AAV (G4) (n = 3). Data are presented as mean ± SD. **p* < 0.05, ***p* < 0.01, ****p* < 0.001, and *****p* < 0.0001.

**Figure S40.** Quantitative analysis of MMP9 protein expression levels in NIH/3T3 cells by Western blotting was performed following co-culture with sKCs. Treatment groups: PBS (G1), AAV (G2), KEV@AAV (G3), SKEV@AAV (G4) (n = 3). Data are presented as mean ± SD. **p* < 0.05, ***p* < 0.01, ****p* < 0.001, and *****p* < 0.0001.

**Figure S41. a.** Western blot showing MMP2 expression in LX-2 cells co-cultured with sTHP-1 cells in a Transwell system. Treatment groups: PBS (G1), AAV (G2), TEV@AAV (G3), STEV@AAV (G4). **b.** Quantification of MMP2 expression in LX-2 cells (n = 3). Data are presented as mean ± SD. **p* < 0.05, ***p* < 0.01, ****p* < 0.001, and *****p* < 0.0001.

**Figure S42. a.** Western blot showing MMP9 expression in LX-2 cells co-cultured with sTHP-1 cells in a Transwell system. Treatment groups: PBS (G1), AAV (G2), TEV@AAV (G3), STEV@AAV (G4). **b.** Quantification of MMP9 expression in LX-2 cells (n = 3). Data are presented as mean ± SD. **p* < 0.05, ***p* < 0.01, ****p* < 0.001, and *****p* < 0.0001.

**Figure S43.** qPCR analysis of *MMP2* and *MMP9* expression in NIH/3T3 cells co-cultured with sKCs in a Transwell system. Treatment groups: PBS (G1), AAV (G2), KEV@AAV (G3), SKEV@AAV (G4) (n = 3). Data are presented as mean ± SD. **p* < 0.05, ***p* < 0.01, ****p* < 0.001, and *****p* < 0.0001.

**Figure S44.** qPCR analysis of *MMP2* and *MMP9* expression in LX-2 cells co-cultured with sTHP-1 cells in a Transwell system. Treatment groups: PBS (G1), AAV (G2), TEV@AAV (G3), STEV@AAV (G4) (n = 3). Data are presented as mean ± SD. **p* < 0.05, ***p* < 0.01, ****p* < 0.001, and *****p* < 0.0001.

**Figure S45.** Quantification of IL-6 and TGF-β in the supernatant of NIH/3T3 fibroblasts following 24-hour drug treatment in a transwell co-culture system with sKCs cells. Treatment groups: PBS (G1), AAV (G2), KEV@AAV (G3), SKEV@AAV (G4) (n = 3). Data are presented as mean ± SD. **p* < 0.05, ***p* < 0.01, ****p* < 0.001, and *****p* < 0.0001.

**Figure S46. a.** Western blot analysis of MMP2 and MMP9 protein expression in NIH/3T3 cells co-cultured with sKCs under indicated treatments. β-actin was used as a loading control. PBS served as a negative control. **b, c.** Quantitative analysis of relative MMP2 (b) and MMP9 (c) expression normalized to β-actin (n = 3). G1–G7 correspond to the treatment groups in order from left to right: PBS (G1), SKEV@AAV (G2), SKEV@AAV + IL-6 (G3), SKEV@AAV + IL-1α (G4), SKEV@AAV + IL-1β (G5), SKEV@AAV + TGF-β (G6), SKEV@AAV + IL-6 + IL-1α + IL-1β + TGF-β (G7). Data are presented as mean ± SD. **p* < 0.05, ***p* < 0.01, ****p* < 0.001, and *****p* < 0.0001.

**Figure S47.** Body weight changes in mice during the treatment period. Groups: PBS (G1), AAV (G2), KEV@AAV (G3), SKEV@AAV (G4) (n = 6).

**Figure S48.** Mice bearing PVTT were treated with PBS, AAV, KEV@AAV, or SKEV@AAV. Liver sections were then subjected to immunofluorescence staining for CD68 (green) and p21 (red), with Dapi (blue) counterstaining for cell nuclei (n = 3). Co-localization of CD68 and p21 indicates sKCs.Scale bar: 20 μm.

**Figure S49. a.** Representative confocal microscopy images showing the expression of CD68 (green) and p21 (red) in mouse normal liver tissues. Nuclei were counterstained with DAPI (blue). Scale bar: 50 µm. **b.** Quantitative analysis of CD68⁺ p21⁺ cells in normal liver tissue (n = 3).

**Figure S50. a.** Representative confocal microscopy images showing the expression of CD68 (green) and p21 (red) in mouse hepatocellular carcinoma tissues. Nuclei were counterstained with Dapi (blue). Scale bar: 50 µm. **b.** Quantitative analysis of CD68⁺ p21⁺ cells in normal liver tissues (n = 3).

**Figure S51.** In vivo IVIS imaging demonstrating Cy5-labeled vectors in mice. Mice were treated with AAV (G1), KEV@AAV (G2), SKEV@AAV (G3) (n = 6).

**Figure S52.** Quantification of bioluminescence intensity in liver tissues across different groups: AAV (G1), KEV@AAV (G2), and SKEV@AAV (G3) (n = 6). Data are presented as mean ± SD. **p* < 0.05, ***p* < 0.01, ****p* < 0.001, and *****p* < 0.0001.

**Figure S53.** Body weight curves of mice during the treatment period (n = 6). PBS (G1), KEV@AAV (G2), SKEV@AAV (G3), PD-1 (G4), KEV@AAV + PD-1 (G5), SKEV@AAV + PD-1 (G6).

**Figure S54.** Quantitative analysis of MMP2 and MMP9 protein expression levels in liver tissues of mice from each treatment group (n = 3). Data are presented as mean ± SD. **p* < 0.05, ***p* < 0.01, ****p* < 0.001, and *****p* < 0.0001.

**Figure S55.** Liver cancer model mice were treated with PBS, AAV, KEV@AAV, or SKEV@AAV. Liver sections were then subjected to immunofluorescence staining for CD68 (green) and p21 (red), with Dapi (blue) counterstaining for cell nuclei (n = 3). Co-localization of CD68 and p21 indicates sKCs. Scale bar: 20 μm.

**Figure S56.** Representative gross anatomy of livers from Hepa 53.4 tumor-bearing mice on day 14 following treatment with different formulations (n = 6). PBS (G1), KEV@AAV (G2), SKEV@AAV (G3), PD-1 (G4), KEV@AAV + PD-1 (G5), SKEV@AAV + PD-1 (G6).

**Figure S57.** Relative TUNEL expression levels across different treatment groups (n = 3). PBS (G1), KEV@AAV (G2), SKEV@AAV (G3), PD-1 (G4), KEV@AAV + PD-1 (G5), SKEV@AAV + PD-1 (G6). Data are presented as mean ± SD. **p* < 0.05, ***p* < 0.01, ****p* < 0.001, and *****p* < 0.0001.

**a**

**b**

**Figure S58. Gating strategy for the identification of macrophages and their phenotypes. a.** The gating strategy for MHC-II⁺ F4/80⁺ cells analysis in vivo. **b.** The gating strategy for M1-phenotype or M2-phenotype macrophage cells analysis in vivo.

**a**

**b**

**Figure S59. Gating strategy for identifying CD8⁺ T cells and Tregs. a.** The gating strategy for CD8⁺ T cells (CD45^+^CD3^+^CD8⁺) analysis in vivo. **b.** The gating strategy for Treg cells (CD45^+^CD4⁺Foxp3⁺) analysis in vivo.

**a**

**b**

**Figure S60. Analysis of memory T cell populations.** **a.** Flow cytometry of effector memory T cells (CD3^+^CD8^+^CD62L^-^CD44^+^) in liver. **b.** Flow cytometry of central memory T cells (CD3^+^CD8^+^CD62L^+^CD44^+^) in blood.

**Figure S61.** H&E staining on major organs (heart, spleen, lung and kidney) of mice after different treatments. PBS (G1), KEV@AAV (G2), SKEV@AAV (G3), PD-1 (G4), KEV@AAV + PD-1 (G5), SKEV@AAV + PD-1 (G6). Scale bar: 100 μm.

**Figure S62.** Serum biochemical indices of mice in each treatment group: alanine aminotransferase, aspartate aminotransferase, blood urea nitrogen, creatinine (n = 3). PBS (G1), KEV@AAV (G2), SKEV@AAV (G3), PD-1 (G4), KEV@AAV + PD-1 (G5), SKEV@AAV + PD-1 (G6). Data are presented as mean ± SD. **p* < 0.05, ***p* < 0.01, ****p* < 0.001, and *****p* < 0.0001.

**Table S1**. qRT-PCR primers used in this study.

| Gene | **Forward (5'-3')** | **Reverse (5'-3')** |
| --- | --- | --- |
| *AAV* | GGAACCCCTAGTGATGGAGTT | CGGCCTCAGTGAGCGA |
| *Cdkn1a* | GTCCAATCCTGGTGATGTCC | GTTTTCGGCCCTGAGATGT |
| *MMP2* | CAGGGCACCTCCTACAACAG | CAGTGGACATAGCGGTCTCG |
| *MMP9* | TAGATCATTCCAGCGTGCCG | GCTTAGAGCCACGACCATACA |

**Table S2**. AAV8-TBG>Kozak-EGFP/mCdkn1a[miR30-shRNA] sequence.

| The sequence of TBG promoter | GGGCTGGAAGCTACCTTTGACATCATTTCCTCTGCGAATGCATGTATAATTTCTACAGAACCTATTAGAAAGGATCACCCAGCCTCTGCTTTTGTACAACTTTCCCTTAAAAAACTGCCAATTCCACTGCTGTTTGGCCCAATAGTGAGAACTTTTTCCTGCTGCCTCTTGGTGCTTTTGCCTATGGCCCCTATTCTGCCTGCTGAAGACACTCTTGCCAGCATGGACTTAAACCCCTCCAGCTCTGACAATCCTCTTTCTCTTTTGTTTTACATGAAGGGTCTGGCAGCCAAAGCAATCACTCAAAGTTCAAACCTTATCATTTTTTGCTTTGTTCCTCTTGGCCTTGGTTTTGTACATCAGCTTTGAAAATACCATCCCAGGGTTAATGCTGGGGTTAATTTATAACTAAGAGTGCTCTAGTTTTGCAATACAGGACATGCTATAAAAATGGAAAGAT |
| --- | --- |
| The sequence of Kozak-EGFP | GCCACCATGGTGAGCAAGGGCGAGGAGCTGTTCACCGGGGTGGTGCCCATCCTGGTCGAGCTGGACGGCGACGTAAACGGCCACAAGTTCAGCGTGTCCGGCGAGGGCGAGGCGGATGCCACCTACGGCAAGCTGACCCTGAAGTTCATCTGCACCACCGGCAAGCTGCCCGTGCCCTGGCCCACCCTCGTGACCACCCTGACCTACGGCGTGCAGTGCTTCAGCCGCTACCCCGACCACATGAAGCAGCACGACTTCTTCAAGTCCGCCATGCCCGAAGGCTACGTCCAGGAGCGCACCATCTTCTTCAAGGACGACGGCAACTACAAGACCCGCGCCGAGGTGAAGTTCGAGGGCGACACCCTGGTGAACCGCATCGAGCTGAAGGGCCATCGACTTCAAGGAGGACGGCAACATCCTGGGGCACAAGCTGGAGTACAACTACAACAGCCACAACGTCTATATCATGG  CCGACAAGCAGAAGAACGGCATCAAGGTGAACTTCAAGATCCGCCACAACATCGAGGACGGCAGCGTGCAGCTCGCCGACCACTACCAGCAGAACACCCCCATCGGCGACGGCCCCGTGCTGCTGCCCGACAACCACTACCTGAGCACCCAGTCCGCCCTGAGCAAAGACCCCAACGAGAAGCGCGATCACATGGTCCTGCTGGAGTTCGTGACCGCCGCCGGGATCACTCTCGGCATGGACGAGCTGTACAAGTAA |
| The sequence of 5'miR-30E/mCdkn1a[miR30-shRNA]/3'miR-30E | TGTTTGAATGAGGCTTCAGTACTTTACAGAATCGTTGCCTGCACATCTTGGAAACACTTGCTGGGATTACTTCGACTTCTTAACCCAACAGAAGGCTCGAGAAGGTATATTGCTGTTGACAGTGAGCGAGCAGACCAGCCTGACAGATTTTAGTGAAGCCACAGATGTAAAATCTGTCAGGCTGGTCTGCCTGCCTACTGCCTCGGACTTCAAGGGGCTAGAATTCGAGCAATTATCTTGTTTACTAAAACTGAATACCTTGCTATCTCTTTGATACATTTTTACAAAGCTGAATTAAAATGGTATAAATTAAATCACTTT |
| shRNA | AGCAGACCAGCCTGACAGATTTTAGTGAAGCCACAGATGTAAAATCTGTCAGGCTGGTCTGCC |

**Table S3.** Particle Size and PDI of Various Formulations.

|  | Particle size(nm) | PDI |
| --- | --- | --- |
| AAV | 17.06 ± 3.498 | 0.199 ± 0.009 |
| KEV | 107.67 ± 0.85 | 0.119 ± 0.030 |
| [KEV@AAV](mailto:KEV@AAV) | 109.63 ± 0.46 | 0.037 ± 0.031 |
| SKEV | 108.73 ± 0.21 | 0.064 ± 0.059 |
| [SKEV@AAV](mailto:SKEV@AAV) | 112.67 ± 3.68 | 0.063 ± 0.049 |

**Source data for all gel electrophoresis and blot results**

Source data for Figure 2D.

Source data for Figure 3Q.

Source data for Figure 3T.

Source data for Figure 6E.

Source data for Figure S11.

Source data for Figure S14.

Source data for Figure S36.

Source data for Figure S37.

**43 kDa**

**92 kDa**

Source data for Figure S41.

Source data for Figure S42.

Source data for Figure S46.
