# Supplementary material for: Targeting p21‐High Senescent Kupffer Cells Nanotherapeutically Potentiates Antitumor Immunity in Advanced Hepatocellular Carcinoma with Portal Vein Tumor Thrombus
Source: Adv Sci (Weinh). 2026 Jul 2:e76384. Online ahead of print. doi: 10.1002/advs.76384 (PMC13336571; doi:10.1002/advs.76384)
Supplement: Supplementary file 2 — Supporting File 2: advs76384‐sup‐0002‐Materials.doc. [file ADVS-9999-e76384-s002.doc]

Revisions to the Supplementary Materials

We have re‑uploaded the Supplementary Materials as a fully revised and consolidated file. All modifications are aligned with the revisions made to the main figures and aim to improve terminological accuracy and overall formatting consistency. No scientific data or statistical conclusions have been altered. The specific revisions are detailed below.

Global unification of terminology and abbreviations: We have thoroughly checked the entire Supplementary Materials file and corrected the following terms throughout (including tables, figure legends, and Methods section):

1.“Edu” or “edu” has been uniformly corrected to “EdU”;

2.“p21” has been changed to lowercase, with italicization (p21) when referring to the gene level, while the protein form remains in normal (non‑italic) font;

3.Mixed uses of “kupffer” and “Kupffer” have been unified to “Kupffer” (capitalized);

4.The abbreviations “KCs” and “sKCs” have been carefully checked to ensure consistent usage throughout the Supplementary file.

5.Standardization of scale bar formats in all figures: For all new and existing images in the Supplementary Materials, we have added a space between each scale bar number and its unit (e.g., “50 μm” instead of “50μm”), and replaced all colons following the scale bars with the English‑style colon (“:”) for consistency.

Consistency of text descriptions: We have simultaneously reviewed the Methods and Results sections in the Supplementary Materials to ensure complete alignment with the main text regarding terminology, abbreviations, and gene/protein nomenclature, with no omissions or discrepancies.

No changes to scientific content: As with the main figures, these revisions are purely formatting and stylistic in nature. None of the original experimental data or statistical outcomes have been affected.

We have replaced the entire Supplementary Materials PDF file with this updated version to ensure that all corrections are properly reflected. Should any further adjustments be required, we are happy to comply.
